# Determination and dietary risk assessment of 284 pesticide residues in local fruit cultivars in Shanghai, China

**DOI:** 10.1038/s41598-021-89204-5

**Published:** 2021-05-06

**Authors:** Yaodan Zhang, Wenshuai Si, Lei Chen, Guoqing Shen, Bing Bai, Changyan Zhou

**Affiliations:** 1 Institute for Agro-Food Standards and Testing Technology, Shanghai Academy of Agricultural Sciences, 1000 Jinqi Road, Shanghai, 201403 China; 2 School of Agriculture and Biology, Shanghai Jiao Tong University, 800 Dongchuan Road, Shanghai, 200240 China

**Keywords:** Risk factors, Chemistry

## Abstract

The presence of pesticide residues has become one of the main risk factors affecting the safety and quality of agro-food. In this study, a multi-residue method for the analysis of 284 pesticides in five local fruit cultivars in Shanghai was developed based on ultrahigh-performance liquid chromatography-quadrupole time-of-flight mass spectrometry (UPLC-QTOF/MS). The limits of determination and the limits of quantitation of pesticides were 0.6–10 and 2–30 μg/kg, respectively. A total of 44, 10, 10, 18, and 7 pesticides were detected in strawberries, watermelons, melons, peaches, and grapes, respectively. The pesticide levels in 95.0% of the samples were below the maximum residual limits (MRLs) prescribed by China, and in 66.2% of the samples below the EU MRLs. The dietary risk assessment study showed big differences in the chronic and acute exposure risk values among different Chinese consumer groups. Through fruit consumption, children/females showed higher exposure risks than adults/males. But both the risk values were less than 100%, indicating that potential dietary risk induced by the pesticides was not significant for Chinese consumers. Nevertheless, certain measures are needed for both growers and the government in order to decrease the MRL-exceeding rate of pesticide residues and ensure the quality and safety of fruits for consumers.

## Introduction

Fruits are important sources of vitamins, minerals, and beneficial phytochemicals, which have important functional properties such as anti-inflammatory, antioxidant, anti-neurodegenerative, and anticarcinogenic activities^1–3^. Besides the nutritive value, fruits can also be a source of pesticide residues. In order to increase fruit yield, a variety of pesticides are used to control pests (e.g., aphids, whiteflies, mites, and nematodes) and diseases (e.g., leaf spot, powdery mildew, gray mold, verticillium wilt, and anthracnose) during the growing season^4–7^. As a result, pesticide residues often occur on the fresh products. According to the Environmental Working Group (EWG)'s 2020 Shopper's Guide to Pesticides in Produce, strawberries have led the ranking of the fresh fruits most contaminated with pesticide residues, followed by nectarines, apples, grapes, and peaches^8^. Residues of some non-recommended and restricted use pesticides can also be found in fruits due to the illegal use or from indirect sources such as soil, plastic films, neighbouring fields and crops, and irrigation waters^9,10^.

Exposure to pesticides may cause acute or chronic toxicity with harmful effects on human health, especially on children and pregnant women who are more vulnerable to the toxic effects^11^. A growing number of epidemiological and molecular studies provide evidence that pesticides have been associated with adverse health effects such as cancers, birth defects, reproductive abnormalities, toxicities, and even death^12–15^. As fruits are mainly consumed fresh, and given the potential hazard of pesticide residues, the European Union (EU) and many other countries have established the maximum residue limits (MRLs) for pesticides in agricultural products to minimize pesticide residue levels and help ensure that pesticides are not overused and residues found in food are tolerable for humans^16–21^. The general MRLs recommended by the EU and China for pesticide residues in fruits are in the range of 0.01–10 mg/kg, depending on the pesticides^18,22^. As the list of banned/restricted and authorized/registered pesticides for use on fruits is continually changing, a sensitive, accurate, and efficient analytical method is necessarily needed to detect a wide diversity of pesticide residues in fruits.

Multi-residue methods incorporate one sample preparation procedure with analytical equipment that is able to determine diverse compounds^17,23^. Chromatographic techniques, such as ultrahigh-performance liquid chromatography (UPLC) and gas chromatography (GC) coupled with tandem mass spectrometry (MS/MS), are widely used for the determination of pesticide residues in food^24^. Gas chromatography is very applicable for non-polar, volatile, and semi-volatile compounds, but not suitable for polar or ionic pesticides^24,25^. LC–MS/MS has become one of the most popular and effective instruments for separation, identification, and quantitation of polar and less volatile pesticides due to its high sensitivity, selectivity, and specificity when operated in multiple reaction monitoring mode^3,25^. Previous studies have reported the widespread use of LC–MS/MS for the determination of pesticide residues in food samples, such as honey^26^, hen eggs^20^, oregano^27^, green tea^28^, fruits and vegetables^7,19,24,29,30^. However, LC–MS/MS method suffers the major disadvantage that the specific masses of the compounds must be predefined before instrumental analysis, and it is limited for the identification of non-target compounds^31,32^. UPLC coupled to high-resolution mass spectrometry is becoming a promising strategy for multi-residue screening of pesticides^33^. Quadrupole time-of-flight mass spectrometry (QTOF/MS) is a hybrid QTOF mass spectrometer with MS/MS capability, which combines the advantages of accurate mass measurement and high resolution^25,34^. QTOF/MS allows high sensitivity and selectivity when acquiring precursor ion and fragment information, and also allows accurate mass determination of both molecular and fragment ions, thus providing high assurance and increased efficiency for the detection, identification, and confirmation of target and non-target compounds^25,31,32^. Several LC-QTOF/MS methods have been reported for the analysis of pesticides, veterinary drugs, and other chemicals in food samples and drinking water^31,32,35–37^. However, it is still a big challenge to screen and quantify a broad range of pesticides simultaneously. The establishment of appropriate analytical methods which can overcome the difficulties such as the complexity of sample and matrix, potential interferences, large number but low concentration of compounds is urgently needed.

In this study, we developed a generic, fast, sensitive, and reliable multi-residue method for the determination of 284 pesticides, based on the pesticides listed in China's latest national food safety standard–maximum residue limits for pesticides in food (GB 2763-2019)^18^, using UPLC-QTOF/MS. The method was used for the analysis of 260 fruit samples collected in different districts of Shanghai to determine exposure levels and to evaluate compliance with MRLs established in Chinese and international regulations. Furthermore, the dietary risk of pesticide residues in the fruits was assessed.

## Results

### Method validation

A rapid method for the determination of 284 pesticides in 260 fruit samples was developed based on UPLC-QTOF/MS (Table S1). The method validation parameters, including linearity, equation, LOD, LOQ, recovery, and RSD, are shown in Table S2. Good linearity was observed for the analytes, with the correlation coefficients (*r*^*2*^) higher than 0.990. The values of LOD and LOQ ranged from 0.6 to 10.0 µg/kg and 2.0 to 30.0 µg/kg, respectively. The recoveries of 269 pesticides (94.7%) ranged from 70 to 120%, with RSDs less than 15.5% (Table S2). For remaining pesticides, 3 compounds (cyromazine, diafenthiuron, and propamocarb) showed recovery values lower than 70% (53–69%), and 12 compounds (bensulfuron-methyl, bifenthrin, chlorsulfuron, ethametsulfuron-methyl, ethoxysulfuron, fenpropidin, florasulam, flumetsulam, maleic hydrazide, metsulfuron-methyl, penoxsulam, and thifensulfuron-methyl) showed recovery values higher than 120% (121–137%), but all the RSD values did not exceed 20% (Table S2). Compared to the chromatograms of spiked sample, no chromatographic peaks close to the retention times of target analytes were found in blank samples, indicating good specificity of the method. For the matrix effect (ME) of the 284 compounds, 81–92% of compounds showed negligible matrix effect (− 20% < ME < 20%), while 5–13% of compounds showed strong matrix suppression and 2–6% showed strong matrix enhancement in blank fruit extracts during UPLC-QTOF/MS analysis (Fig. S1).

### Pesticide residues in fruit samples

**Figure 1:**
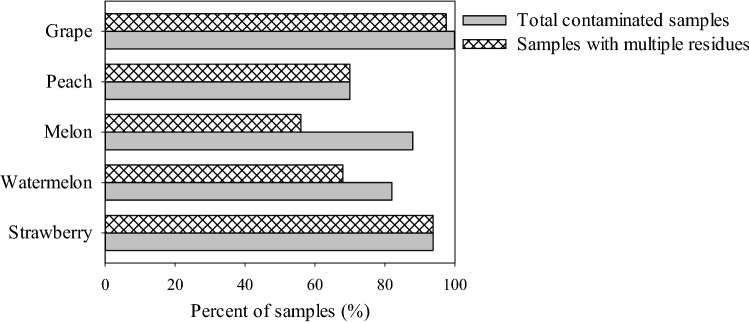
Percent of contaminated samples in strawberries, watermelons, melons, peaches, and grapes.

The developed method was applied to the analysis of 284 pesticides in 260 fruit samples. Matrix-matched calibration curves were used to calculate the concentrations of pesticides in fruits. Pesticide residues were detected in 228 samples (87.7% of the total), mainly fungicides, insecticides and acaricides. The detection rates of pesticide residues in strawberry, watermelon, melon, peach, and grape samples were 93.7%, 82%, 88%, 70%, and 100%, respectively, and more than 56% of the samples contained at least two of the analyzed pesticide residues (Fig. 1). Detailed data of the pesticide residues detected in the fruit samples are shown in Table 1.

**Table 1:** Occurrence of pesticide residues in fruit samples.

Fruit	Pesticide	Min–Max (µg/kg)	Mean (µg/kg)	China MRL (µg/kg)	EU MRL (µg/kg)	No. of samples > China MRL	No. of samples > EU MRL
Strawberry (n = 80)	Acetamiprid	4.6–87.2	19.9	2000	500	0	0
	Azoxystrobin	4.1–148.3	40.1	10,000	10,000	0	0
	Bifenazate	32.2–540.1	151.4	2000	3000	0	0
	Boscalid	4.5–1823.8	207.5	3000	6000	0	0
	Carbendazim	3.4–294.8	58.5	500	100**	0	2 (2.5%)
	Chlorantraniliprole	8.6–38.5	15.9	1000*	1000	0	0
	Cyantraniliprole	18.7–69.6	45.5	4000*	1500	0	0
	Cyprodinil	3.8–624.5	198.3	2000	5000	0	0
	Diethyl aminoethyl hexanoate	20.0–22.8	21.2	–	–	–	–
	Difenoconazole	5.3–167.6	28.1	–	2000	–	0
	Dimethomorph	12.3–33.8	23.0	50	700	0	0
	Dinotefuran	5.8–44.6	25.2	–	–	–	–
	Ethirimol	5.6–665.2	131.1	–	200	–	5 (6.3%)
	Etoxazole	4.9–29.9	15.8	–	200	–	0
	Flonicamid	5.0–126.9	34.7	–	30**	–	12 (15.0%)
	Fludioxonil	9.3–248.4	53.7	3000	4000	0	0
	Fluopyram	3.3–6260.5	544.9	400*	2000	13 (16.3%)	4 (5.0%)
	Hexaconazole	15.6–24.7	20.2	–	10**	–	2 (2.5%)
	Imazalil	7.8–163.1	85.4	2000	2000	0	0
	Indoxacarb	4.0–7.5	5.9	–	600	–	0
	Kresoxim-methyl	7.9–58.4	20.6	2000	1500	0	0
	Metalaxyl	6.3–11.5	9.8	–	600	–	0
	Methoxyfenozide	7.2–7.4	7.3	2000	2000	0	0
	Myclobutanil	9.3–447.2	126.1	1000	1000	0	0
	Nitenpyram	4.9–15.3	10.1	–	–	–	–
	Novaluron	11.0–26.7	18.9	500	500	0	0
	Oxadixyl	3.5–7.1	5.3	–	10**	–	0
	Prochloraz	6.1–96.9	34.3	–	30**	–	2 (2.5%)
	Propamocarb	3.8–67.0	29.8	–	10**	–	3 (3.8%)
	Pyraclostrobin	3.6–681.8	82.5	2000	1500	0	0
	Pyrimethanil	6.8–2193.3	243.9	7000	5000	0	0
	Pyriproxyfen	7.7	7.7	–	50**	–	0
	Spinetoram A	10.8–20.3	16.7	–	200	–	0
	Spirodiclofen	11.9	11.9	2000	2000	0	0
	Spirotetramat^#^	8.3–242.9	69.7	1500*	400	0	0
	Sulfoxaflor	12.0–203.0	78.3	500*	500	0	0
	Tebuconazole	12.0–16.0	13.4	–	20**	–	0
	Thiacloprid	4.5–36.5	15.9	1000	1000	0	0
	Thiamethoxam	3.8–17.1	8.2	500	300	0	0
	Thiophanate-methyl	6.8–1124.5	112.4	–	100**	–	2 (2.5%)
	Triadimefon	7.1–17.0	12.0	700	10**	0	1 (1.3%)
	Triadimenol	77.8	77.8	700	500	0	0
	Trichlorfon	7.4	7.4	200	10**	0	0
	Trifloxystrobin	3.4–672.4	88.3	1000	1000	0	0
Watermelon (n = 50)	Acetamiprid	3.6–32.0	18.1	200	200	0	0
	Clothianidin	2.3–13.9	7.7	–	20**	–	0
	Dinotefuran	15.3–121.8	57.2	1000	–	0	–
	Ethirimol	2.4–54.6	21.4	–	80	–	0
	Etoxazole	2.5–4.4	3.5	–	50	–	0
	Methoxyfenozide	6.5–15.2	9.7	–	10**	–	3 (6.0%)
	Nitenpyram	2.3–7.2	3.5	–	–	–	–
	Oxadixyl	5.8–19.2	11.5	–	10**	–	6 (12.0%)
	Pymetrozine	3.6–8.9	5.9	–	300	–	0
	Pyraclostrobin	2.7	2.7	500	500	0	0
Melon (n = 50)	Azoxystrobin	2.1–27.6	12.8	–	1000	–	0
	Difenoconazole	3.9–12.1	6.8	700	200	0	0
	Ethirimol	7.3–49.9	24.4	–	80	–	0
	Fluopyram	7.2–516.8	210.9	–	400	–	3 (6.0%)
	Fosthiazate	3.1–88.7	26.0	–	20**	–	11 (22.0%)
	Indoxacarb	3.3–6.8	4.6	–	500	–	0
	Paclobutrazol	4.0–10.9	8.3	–	10**	–	1 (2.0%)
	Pyraclostrobin	2.6–26.4	9.9	500	500	0	0
	Thiamethoxam	3.3–5.9	4.4	–	150	–	0
	Trifloxystrobin	3.3–11.4	8.0	–	300	–	0
Peach (n = 40)	Acetamiprid	18.4–294.2	89.6	2000	200	0	2 (5.0%)
	Carbendazim	4.8–689.6	123.3	2000	200	0	3 (7.5%)
	Clothianidin	2.9–74.5	19.1	200	150	0	0
	Cyhalothrin	6.9–64.7	16.8	500	150	0	0
	Cypermethrin	15.2–70.7	34.2	1000	2000	0	0
	Difenoconazole	3.3–146.3	36.6	500	500	0	0
	Hexaflumuron	10.8–118.1	38.1	–	–	–	–
	Imidacloprid	3.3–151.4	41.9	500	500	0	0
	Indoxacarb	4.3	4.3	1000	1000	0	0
	Nitenpyram	4.2	4.2	–	–	–	–
	Paclobutrazol	4.3–281.8	124.9	–	150	–	3 (7.5%)
	Pymetrozine	4.4–13.7	7.7	–	30	–	0
	Pyraclostrobin	2.4–4.6	3.3	1000	300	0	0
	Pyridaben	7.8	7.8	–	300	–	0
	Pyriproxyfen	3.0–7.5	5.2	–	500	–	0
	Spinosad A	9.2	9.2	200*	600	0	0
	Spirodiclofen	12.1–290.6	79.3	2000	2000	0	0
	Thiophanate-methyl	7.1–50.2	19.0	–	2000	–	0
Grape (n = 40)	Difenoconazole	11.1–408.0	106.3	500	3000	0	0
	Ethirimol	71.0–677.1	309.2	–	500	–	2 (5.0%)
	Forchlorfenuron	2.3–3.8	2.9	50	10**	0	0
	Methoxyfenozide	9.9	9.9	1000	1000	0	0
	Picoxystrobin	4.4–194.6	92.8	1000	10**	0	3 (7.5%)
	Pyraclostrobin	31.3–356.2	152.5	2000	1000	0	0
	Triflumuron	12.9–1619.7	468.0	–	10**	–	40 (100%)

^#^Sum of spirotetramat and spirotetramat-enol, expressed as spirotetramat.

*Temporary MRLs prescribed by the National Standard of China.

**Lower limit of analytical determination prescribed by the EU.

**Figure 2:**
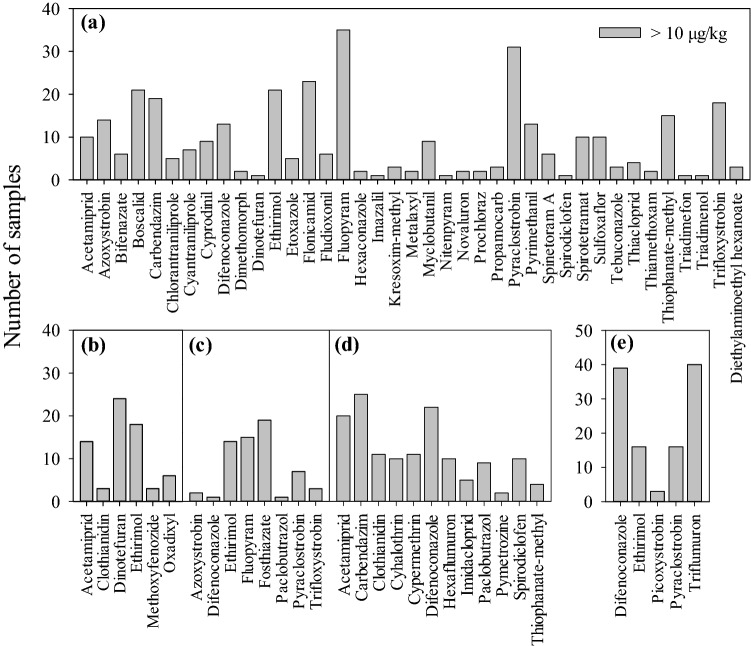
Number of samples with different pesticide residues in strawberries (**a**), watermelons (**b**), melons (**c**), peaches (**d**), and grapes (**e**).

A total of 44, 10, 10, 18, and 7 pesticides were detected in strawberry, watermelon, melon, peach, and grape samples, respectively (Table 1). The pesticides with the highest detection frequency were fluopyram (3.3–6260.5 µg/kg), pyraclostrobin (3.6–681.8 µg/kg), and flonicamid (5.0–126.9 µg/kg) in strawberries; dinotefuran (15.3–121.8 µg/kg), ethirimol (2.4–54.6 µg/kg), and acetamiprid (3.6–32.0 µg/kg) in watermelons; fosthiazate (3.1–88.7 µg/kg), fluopyram (7.2–516.8 µg/kg), and ethirimol (7.3–49.9 µg/kg) in melons; carbendazim (4.8–689.6 µg/kg), difenoconazole (3.3–146.3 µg/kg), and acetamiprid (18.4–294.2 µg/kg) in peaches; and triflumuron (12.9–1619.7 µg/kg), difenoconazole (11.1–408.0 µg/kg), and ethirimol (71.0–677.1 µg/kg) in grapes (Fig. 2a–e, Table 1). The levels of fluopyram residues in 13 strawberry samples (16.3%) were exceeded the Chinese MRL, and in 4 strawberry (5.0%) and 3 melon samples (6.0%) were exceeded the EU MRL (Table 1). In addition, the levels of carbendazim, ethirimol, flonicamid, hexaconazole, prochloraz, propamocarb, thiophanate-methyl, and triadimefon residues in at least one strawberry sample; methoxyfenozide and oxadixyl in at least three watermelon samples; fosthiazate and paclobutrazol in at least one melon sample; acetamiprid, carbendazim, and paclobutrazol in at least two peach samples; and ethirimol, picoxystrobin, and triflumuron in at least two grape samples were exceeded the EU MRLs or exceeded the lower limit of analytical determination prescribed by the EU (Table 1).

### Chronic and acute dietary exposure assessment

A total of 57 pesticide residues were detected in all the fruit samples. The results of chronic and acute dietary exposure risk assessment of the pesticide residues in fruit samples are shown in Tables 2 and 3.

**Table 2:** Chronic dietary exposure assessment of detected pesticide residues in fruits among different Chinese consumer groups.

Pesticide	ADI (mg/kg d)	%ADI					
		2–4 Male	2–4 Female	18–30 Male	18–30 Female	60–70 Male	60–70 Female
Acetamiprid	0.07	0.5650	0.6040	0.1259	0.1833	0.1005	0.1168
Azoxystrobin	0.2	0.0820	0.0876	0.0183	0.0266	0.0146	0.0170
Bifenazate	0.01	4.6923	5.0165	1.0460	1.5226	0.8348	0.9703
Boscalid	0.04	1.6078	1.7188	0.3584	0.5217	0.2860	0.3325
Carbendazim	0.03	1.8782	2.0079	0.4187	0.6095	0.3341	0.3884
Chlorantraniliprole	2	0.0025	0.0026	0.0005	0.0008	0.0004	0.0005
Clothianidin	0.1	0.0831	0.0888	0.0185	0.0270	0.0148	0.0172
Cyantraniliprole	0.03	0.4701	0.5025	0.1048	0.1525	0.0836	0.0972
Cyhalothrin	0.02	0.2603	0.2783	0.0580	0.0845	0.0463	0.0538
Cypermethrin	0.02	0.5300	0.5666	0.1181	0.1720	0.0943	0.1096
Cyprodinil	0.03	2.0486	2.1902	0.4567	0.6648	0.3645	0.4236
Diethyl aminoethyl hexanoate	0.023	0.2857	0.3054	0.0637	0.0927	0.0508	0.0591
Difenoconazole	0.01	5.5105	5.8913	1.2284	1.7881	0.9804	1.1395
Dimethomorph	0.2	0.0356	0.0381	0.0079	0.0116	0.0063	0.0074
Dinotefuran	0.2	0.1277	0.1365	0.0285	0.0414	0.0227	0.0264
Ethirimol	0.035	4.3045	4.6019	0.9596	1.3968	0.7658	0.8901
Etoxazole	0.05	0.1196	0.1279	0.0267	0.0388	0.0213	0.0247
Flonicamid	0.07	0.1536	0.1643	0.0342	0.0499	0.0273	0.0318
Fludioxonil	0.4	0.0416	0.0445	0.0093	0.0135	0.0074	0.0086
Fluopyram	0.01	23.4244	25.0429	5.2219	7.6011	4.1674	4.8438
Forchlorfenuron	0.07	0.0128	0.0137	0.0029	0.0042	0.0023	0.0027
Fosthiazate	0.004	2.0145	2.1537	0.4491	0.6537	0.3584	0.4166
Hexaconazole	0.005	1.2521	1.3386	0.2791	0.4063	0.2228	0.2589
Hexaflumuron	0.02	0.5904	0.6312	0.1316	0.1916	0.1050	0.1221
Imazalil	0.03	0.8823	0.9432	0.1967	0.2863	0.1570	0.1824
Imidacloprid	0.06	0.2164	0.2314	0.0482	0.0702	0.0385	0.0448
Indoxacarb	0.01	0.4587	0.4904	0.1023	0.1488	0.0816	0.0949
Kresoxim-methyl	0.4	0.0160	0.0171	0.0036	0.0052	0.0028	0.0033
Metalaxyl	0.08	0.0380	0.0406	0.0085	0.0123	0.0068	0.0079
Methoxyfenozide	0.1	0.0834	0.0891	0.0186	0.0271	0.0148	0.0172
Myclobutanil	0.03	1.3027	1.3927	0.2904	0.4227	0.2318	0.2694
Nitenpyram	0.53	0.0104	0.0111	0.0023	0.0034	0.0019	0.0022
Novaluron	0.01	0.5858	0.6262	0.1306	0.1901	0.1042	0.1211
Oxadixyl	0.01	0.5207	0.5567	0.1161	0.1690	0.0926	0.1077
Paclobutrazol	0.1	0.4128	0.4413	0.0920	0.1340	0.0734	0.0854
Picoxystrobin	0.09	0.3196	0.3417	0.0712	0.1037	0.0569	0.0661
Prochloraz	0.01	1.0631	1.1365	0.2370	0.3450	0.1891	0.2198
Propamocarb	0.4	0.0231	0.0247	0.0051	0.0075	0.0041	0.0048
Pymetrozine	0.03	0.1405	0.1502	0.0313	0.0456	0.0250	0.0291
Pyraclostrobin	0.03	2.5920	2.7711	0.5778	0.8411	0.4611	0.5360
Pyridaben	0.01	0.2417	0.2584	0.0539	0.0784	0.0430	0.0500
Pyrimethanil	0.2	0.3780	0.4041	0.0843	0.1226	0.0672	0.0782
Pyriproxyfen	0.1	0.0400	0.0427	0.0089	0.0130	0.0071	0.0083
Spinetoram A	0.05	0.1035	0.1107	0.0231	0.0336	0.0184	0.0214
Spinosad A	0.02	0.1426	0.1524	0.0318	0.0463	0.0254	0.0295
Spirodiclofen	0.01	2.8266	3.0219	0.6301	0.9172	0.5029	0.5845
Spirotetramat	0.05	0.4320	0.4619	0.0963	0.1402	0.0769	0.0893
Sulfoxaflor	0.05	0.4853	0.5189	0.1082	0.1575	0.0863	0.1004
Tebuconazole	0.03	0.1384	0.1480	0.0309	0.0449	0.0246	0.0286
Thiacloprid	0.01	0.4928	0.5268	0.1099	0.1599	0.0877	0.1019
Thiamethoxam	0.08	0.0488	0.0522	0.0109	0.0158	0.0087	0.0101
Thiophanate-methyl	0.09	0.4525	0.4838	0.1009	0.1468	0.0805	0.0936
Triadimefon	0.03	0.1240	0.1325	0.0276	0.0402	0.0221	0.0256
Triadimenol	0.03	0.8037	0.8593	0.1792	0.2608	0.1430	0.1662
Trichlorfon	0.002	1.1467	1.2260	0.2556	0.3721	0.2040	0.2371
Trifloxystrobin	0.04	0.7462	0.7977	0.1663	0.2421	0.1327	0.1543
Triflumuron	0.014	10.3605	11.0763	2.3096	3.3619	1.8432	2.1424

**Table 3:** Acute dietary exposure assessment of detected pesticide residues in fruits among different Chinese consumer groups.

Pesticide	ARfD (mg/kg d)	%ARfD					
		2–4 Male	2–4 Female	18–30 Male	18–30 Female	60–70 Male	60–70 Female
Acetamiprid	0.1	9.9507	10.4705	3.4861	4.0097	3.4406	3.8842
Carbendazim	0.5	4.7391	4.9866	1.6603	1.9097	1.6386	1.8499
Clothianidin	0.6	0.3546	0.3731	0.1242	0.1429	0.1226	0.1384
Cyhalothrin	0.02	7.7869	8.1937	2.7281	3.1378	2.6925	3.0396
Cypermethrin	0.04	4.2545	4.4768	1.4905	1.7144	1.4711	1.6607
Difenoconazole	0.3	5.8890	6.1966	2.0631	2.3730	2.0362	2.2987
Dimethomorph	0.6	0.1356	0.1427	0.0475	0.0546	0.0469	0.0529
Dinotefuran	1	0.4005	0.4214	0.1403	0.1614	0.1385	0.1563
Fluopyram	0.5	32.6272	34.3316	11.4307	13.1474	11.2815	12.7358
Imazalil	0.05	7.8510	8.2611	2.7505	3.1636	2.7146	3.0646
Imidacloprid	0.4	0.9111	0.9587	0.3192	0.3671	0.3150	0.3556
Indoxacarb	0.1	0.4468	0.4701	0.1565	0.1800	0.1545	0.1744
Methoxyfenozide	0.9	0.0870	0.0915	0.0305	0.0351	0.0301	0.0340
Picoxystrobin	0.09	5.2043	5.4762	1.8233	2.0971	1.7995	2.0315
Prochloraz	0.1	2.3315	2.4533	0.8168	0.9395	0.8062	0.9101
Propamocarb	2	0.0806	0.0849	0.0283	0.0325	0.0279	0.0315
Pymetrozine	0.1	0.5445	0.5730	0.1908	0.2194	0.1883	0.2125
Pyraclostrobin	0.05	51.5945	54.2898	18.0757	20.7905	17.8398	20.1396
Spirotetramat	1	0.5846	0.6151	0.2048	0.2356	0.2021	0.2282
Sulfoxaflor	0.3	1.6290	1.7141	0.5707	0.6564	0.5632	0.6359
Tebuconazole	0.3	0.1282	0.1349	0.0449	0.0517	0.0443	0.0500
Thiacloprid	0.03	2.9286	3.0816	1.0260	1.1801	1.0126	1.1432
Thiamethoxam	1	0.0553	0.0582	0.0194	0.0223	0.0191	0.0216
Triadimefon	0.08	0.5106	0.5373	0.1789	0.2058	0.1766	0.1993
Triadimenol	0.08	2.3409	2.4632	0.8201	0.9433	0.8094	0.9138

For the chronic dietary exposure assessment, ADI values were obtained from the National Standard of China^18^. In different age and sex groups, the risk of chronic dietary intake (%ADI) of the 57 pesticides from eating the fruits was less than 100% (Table 2). The risk values of fluopyram and triflumuron were the highest, especially in the 2–4 year old children groups (%ADI > 10%), followed by difenoconazole, bifenazate, and ethirimol (Table 2). Among the different Chinese population groups, the risk of chronic dietary intake of the pesticides was in the following order: 2–4 year old female children > 2–4 year old male children > 18–30 year old female adult > 18–30 year old male adult > 60–70 year old female adult > 60–70 year old male adult.

For the acute dietary exposure assessment, ARfD values were obtained from the joint Food and Agriculture Organization of the United Nations (FAO) and World Health Organization (WHO) Meeting on Pesticide Residues (JMPR)^38^. A list of pesticides has been evaluated by the JMPR, and it has decided that ARfD values were unnecessary for 21 pesticides, including azoxystrobin, bifenazate, boscalid, chlorantraniliprole, cyantraniliprole, cyprodinil, etoxazole, flonicamid, fludioxonil, fosthiazate, kresoxim-methyl, metalaxyl, myclobutanil, novaluron, pyrimethanil, pyriproxyfen, spinetoram A, spinosad A, spirodiclofen, thiophanate-methyl, and trifloxystrobin^38^. In addition, there are no authorized/established ARfD values for diethyl aminoethyl hexanoate, ethirimol, forchlorfenuron, hexaconazole, hexaflumuron, nitenpyram, oxadixyl, paclobutrazol, pyridaben, trichlorfon, and triflumuron^38,39^, and the corresponding risk index could not be computed. Thus, the risk values of acute dietary exposure (%ARfD) of 25 pesticides are given in Table 3. All the %ARfD values were lower than 100% in all the consumption groups. The risk of acute intake in different groups was in the order of 2–4 year old female children > 2–4 year old male children > 18–30 year old female adult > 60–70 year old female adult > 18–30 year old male adult > 60–70 year old male adult. Pyraclostrobin, fluopyram, and acetamiprid exhibited higher acute risks for Chinese consumers than other pesticides, their %ARfD values were 17.8398–54.2898%, 11.2815–34.3316%, and 3.4406–10.4705%, respectively.

## Discussion

The linearity (> 0.990) was considered acceptable^40^. Most of the LOQ values (82.4%) were below the non-detectable default value (0.01 mg/kg) recommended in the EU regulations^16^. The obtained LOQs were much lower than the Chinese and EU MRLs (0.01–10 mg/kg) for pesticides in strawberries, watermelons, melons, peaches, and grapes^18,22^, indicating that the developed method is sensitive and suitable for comprehensive monitoring of pesticide residues in the fruit samples. The SANTE guidelines recommend that the acceptable mean recoveries are those within the range 70–120%, with an associated RSD ≤ 20%^41^. The accuracy and precision obtained in this study are comparable with those reported in previous studies. Yang et al.^32^ determined 50 pesticides in starfruit and Indian jujube using LC-QTOF/MS, and obtained recoveries between 63 and 119%, with RSDs of 0.2–3.2%. Sivaperumal et al.^31^ achieved satisfactory recoveries ranging from 74 to 111%, with RSDs below 13.2%. Matrix effect is the combined effect of all components of the sample other than the analytes on the measurement, which can comprehensively suppress or enhance the response of the target compounds^28,33^. The values of matrix effect between –20% and 20% are considered acceptable^33^. Matrix ionization suppression still existed in pesticide analysis, which is in accordance with the results of earlier studies^26,28,32^.

Multiple pesticide residues in fruits are commonly observed. Li et al.^42^ found that carbendazim, cyhalothrin, acetamiprid, cypermethrin, imidacloprid, as well as difenoconazole had high detection frequency in peaches. Previous studies have also noted that pesticides, especially fungicides and insecticides such as carbendazim, pyrimethanil, trifloxystrobin, and acetamiprid, had high detection frequency in strawberry fruits in China, Poland, and Turkey^1,30,43^. In this study, the strawberry samples were collected in January when strawberries first appeared on market. In order to increase strawberry yield and maximize returns, growers apply high levels of various pesticides during this period. Chu et al.^30^ also noted that the detection rates of pesticides in strawberries collected in January were higher than that in strawberries collected in other months.

MRLs are the maximum permissible values of pesticide residues in food. They are established to ensure the proper use of pesticides in agriculture and reduce harmful pesticide intake in humans, and thus protect human health^17^. The overall result revealed that 95.0% of samples were below the MRLs prescribed by the National Standard of China^18^, and 66.2% of samples were below the MRLs prescribed by the EU^16^. Currently, there are many pesticides which are registered for use on fruits in China (China Pesticide Information Network. http://www.icama.org.cn/hysj/index.jhtml). In this study, only 11, 3, 1, 5, and 4 detected pesticides are registered pesticides in strawberries, watermelons, melons, peaches, and grapes, respectively. Moreover, some pesticides had no corresponding residue limits authorized in Chinese regulations but had high detection rates, such as ethirimol in strawberries, watermelons, melons, and grapes, fosthiazate and fluopyram in melons, and triflumuron in grapes. This is a great challenge for the government to monitor the use of pesticides.

The results of chronic and acute dietary exposure assessment are in agreement with the results of Chu et al.^30^. Similar results from studies on chronic dietary risk of pesticides in fruits in Poland and in peaches in China have also been reported^1,42^. They also indicated that the chronic risk values for children were higher than that for adults, but neither exceeded 100%. Through fruit consumption, children had higher chronic and acute exposure risks than adults, and females had higher exposure risks than males, which supports previous findings^30,44,45^. In our study, the evaluated fruits exhibited an acceptably low risk to Chinese consumers, however, other studies indicated that some kind of pesticide residues in fruits showed unacceptable acute risks, especially for infants and children^42,44,45^. Although pesticides with high detection rates do not mean high exposure risks, the potential risks should be paid more attention to the pesticides with high levels (> MRLs) and the pesticides with no regulations for ARfD values. For example, ethirimol showed relatively high detection rates in the fruits, and the levels exceeded the EU MRLs in some strawberry and grape samples, and triflumuron was detected in all the grape samples and showed relatively high chronic dietary risk, but we could not evaluate their acute dietary risks. Such a risk cannot be excluded, especially for children and females.

In order to decrease the MRL-exceeding rate of pesticide residues in fruits, certain measures, such as increased education of growers, control of the sale and use of pesticides, rigorous monitoring of pesticides before harvest, implementation of integrated pest management methods, as well as improvement of regulations, are urgently needed^17,43^. Both growers and government are responsible for food safety, the applications and monitoring programs for pesticides in domestic products must be responsibly carried out.

## Materials and methods

### Chemicals and reagents

High-performance liquid chromatography (HPLC) grade methanol and acetonitrile were obtained from Merck (Darmstadt, Germany), ammonium acetate (CH_3_COONH_4_) was purchased from ANPEL Laboratory Technologies Inc. (Shanghai, China). Analytical grade sodium chloride (NaCl) was purchased from Shanghai Titan Scientific Co., Ltd. (Shanghai, China). Ultrapure water was prepared using a Milli-Q purification system (Millipore, Billerica, MA, USA).

Twenty-one groups of certified pesticide mixed standard solutions (100 µg/mL in HPLC-grade acetonitrile, methanol, toluene, hexane, and acetone) were purchased from Alta Scientific Co., Ltd (Tianjin, China). These stock solutions were stored at –20 ℃ in the dark. From each stock solution, a mixed standard working solution contained all the pesticides was prepared at 1 µg/mL by appropriate dilution with acetone, which was stored at 4 ℃ and renewed every 2 months.

### Sample preparation

A total of 260 fruit samples, including five local cultivars: strawberry (*Fragaria* × *ananassa* Duch.), watermelon (*Citrullus lanatus* (Thunb.) Mansfeld), melon (*Cucumis melo* L.), peach (*Prunus persica* (L.) Batsch), and grape (*Vitis vinifera* L.), were collected from different fields in different districts of Shanghai, China (Table S3) in 2019 and 2020. All the fruit samples were collected with the permission of the farmers. The wet weight for one sample was 3 kg for strawberry, melon, peach, grape, and tomato, and each watermelon sample include three watermelons. All samples were kept cool and the whole fruits of strawberries, watermelons, melons, grapes, and peached (without peach pit) were homogenized in a commercial blender, and then stored at –20 ℃ in the dark until chemical analysis. For peaches, the weight of the peach pit was included when calculating the pesticide residues.

Pesticides in fruit samples were extracted using a modified method of Yang et al.^32^. A blended sample (around 10 g) was weighed into a 50 mL polypropylene centrifuge tube, and mixed thoroughly with 10 mL acetonitrile for 20 min using an advanced multi-tube vortexer (Troemner LLC., Thorofare, NJ, USA). Subsequently, in order to improve the extraction efficiency, 5 g of NaCl was added to the tube and vortexed for 1 min. The mixture was centrifuged at 5000 rpm for 5 min in a Thermo Fisher ST 16R centrifuge (Osterode, Germany). After centrifugation, 1 mL of the supernatant was transferred to a 10 mL glass tube, 1 mL of Milli-Q water was added, and the tube was vortexed for 30 s. The extract was then filtered through a 0.22 µm nylon syringe filter (Pall Corp., Port Washington, NY, USA) and 3 μL were injected into the UPLC-QTOF/MS system.

Strawberry, watermelon, melon, peach, and grape fruit samples without pesticide residues detected were used as blank samples. Pesticide-spiked blank samples were used as quality control samples. The blank and quality control samples were extracted simultaneously with the fruit samples by the same method. Pesticide residues were quantified by the external standard calibration curve method. The samples were diluted when the pesticide content exceeded the standard calibration curve range. The limit of detection (LOD) and the limit of quantification (LOQ) of the method were defined as 3 and 10 times of spiked blank samples’ signal-to-background noise (S/N), respectively. Samples were run in the following order: solvent (acetonitrile/water 1/1, v/v)—calibration curves—blank samples—quality control samples—solvent—fruit samples—calibration curves—solvent. The blank and quality control samples were inserted every 20 fruit samples.

### UPLC-QTOF/MS analysis

The pesticides were identified and quantified using an ultrahigh-performance liquid chromatography system (Waters Acquity I-Class, Waters Corporation, Milford, MA, USA) coupled to a quadrupole time-of-flight mass spectrometer (AB SCIEX TripleTOF 5600+, Framingham, MA, USA). The chromatographic conditions, including the selection of chromatographic column, mobile phase and buffer solution, and the gradient elution program, were optimized to achieve good separation. The chromatographic separation was performed on a Waters ACQUITY UPLC HSS T3 column (2.1 × 100 mm; particle size 1.8 µm, Waters, Ireland) with a flow rate of 0.4 mL/min, and the column temperature was kept at 45 ℃. Mobile phases were 100% methanol (solvent A) and 5 mM ammonium acetate in Milli-Q water (solvent B). The gradient for solvent A was as follows: 0–0.5 min, 2%; 0.5–15 min, 2–98%; 15–17 min, 98%; 17–17.1 min, 98–2%; 17.1–20 min, 2%. The injection volume was 3 μL.

The QTOF/MS spectra were acquired in positive electrospray ionization mode (ESI^+^) with the following parameters: mass range, 50–1000 m*/z*; ionspray voltage floating (ISVF), 5500 V; temperature (TEM), 500 ℃; ion source gas (GS1) nebulizer gas pressure, 50 psi; ion source gas (GS2) auxiliary heater gas pressure, 50 psi; curtain gas (CUR), 35 psi; declustering potential (DP), 80 V; collision energy (CE), 35 ± 15 eV. The mass spectrometry analysis was conducted in full scan TOF/MS mode and in MS/MS mode. Detailed instrument conditions are described in Yang et al.^32^.

### Method validation

The fruit samples were analyzed by UPLC-QTOF/MS in advance, and the sample detected as pesticide-free was used as the blank matrix sample for the spiking experiment. The validation parameters included linearity, sensitivity, accuracy, precision, specificity, and matrix effect. The linearity was determined using matrix-matched calibration curves, which were obtained by adding mixed pesticide standard solution into the extract of blank matrix at seven concentration levels in the range of 2–200 µg/kg, analyzed in triplicate. The sensitivity was assessed by LODs and LOQs. Method accuracy was evaluated by recovery studies. The blank matrix sample was spiked at two concentration levels (10 and 100 µg/kg) with six replicates for each level, then the spiked samples were extracted according to the procedure as described in** Section *Sample preparation*. The relative standard deviation (RSD) of the pesticides from the recovery studies were used to evaluate the precision. To assess the specificity, the chromatograms of blank sample and spiked sample at LOD levels were analyzed. The S/N ratios of chromatographic peaks in blank sample had to be lower than that in spiked sample^40^. The matrix effect (ME) was evaluated by comparing the signal intensity of matrix-matched standard with pure solvent standard at the same concentration^28,33^. ME (%) was calculated based on the following equation^28^: ME (%) = (peak area of matrix-matched standard − peak area of solvent standard)/ peak area of solvent standard × 100%.

### Dietary exposure risk assessment

The chronic and acute dietary exposure risk values were determined by comparing the value of national estimated daily intake (NEDI) of pesticides with acceptable daily intake (ADI), and by comparing the value of estimated short-term intake (ESTI) of pesticides with acute reference dose (ARfD), respectively, according to the following Eqs. ^30,46,47^.

1 $$ {\text{NEDI }} = \, \left( {\sum {\text{R }} \times {\text{ F}}} \right)/{\text{bw}} $$

2 $$ \% {\text{ADI }} = \, \left( {{\text{NEDI}}/{\text{ADI}}} \right) \, \times { 1}00\% $$

The chronic risk was calculated using the above Eqs. (1) and (2). NEDI (mg/kg·d) indicates the national estimated daily intake; R (mg/kg) is the mean amount of pesticide residues in fruit samples; F (kg/d) is the dietary consumption of fruits in China; bw (kg) is the average body weight; ADI (mg/kg·d) is the acceptable daily intake.

3 $$ {\text{ESTI }} = \, \left( {\sum {\text{HR }} \times {\text{ LP}}} \right)/{\text{bw}} $$

4 $$ \% {\text{ARfD }} = \, \left( {{\text{ESTI}}/{\text{ARfD}}} \right) \, \times { 1}00\% $$

The acute risk was calculated using the above Eqs. (3) and (4). ESTI (mg/kg·d) represents the estimated short-term intake; HR (mg/kg) is the highest amount of pesticide residues in fruit samples; LP (kg/d) is the large portion of fruit consumption in Chinese population; ARfD (mg/kg·d) is the acute reference dose.

In this study, fruit consumption group was divided into three sensitive population groups, including 2–4, 18–30, and 60–70 year old male and female groups. The average body weight and fruit consumption in different groups in China are shown in Table S4. If %ADI or %ARfD value is lower than 100%, the exposure risk is acceptable. The higher the value, the greater the risk. While when the value is higher than 100%, it indicates an unacceptably high risk to consumersy^30,46^.

### Approvals and permissions

This study was approved by Shanghai Municipal Agriculture and Rural Affairs Committee (Approval number: 2019-02-08-00-12-F01144). The experiment was performed in accordance with the regulations (NY/T 789-2004) established by the Ministry of Agriculture and Rural Affairs of the People’s Republic of China. All the farms or farmer professional cooperatives are legally registered in Shanghai.

## Supplementary Information

Supplementary Information.

## Data Availability

All data generated and/or analyzed during the current study are available from the corresponding author on reasonable request.

## References

[1] Szpyrka E (2015). Evaluation of pesticide residues in fruits and vegetables from the region of south-eastern Poland. Food Control.

[2] Méndez-Lagunas L, Rodríguez-Ramírez J, Cruz-Gracida M, Sandoval-Torres S, Barriada-Bernal G (2017). Convective drying kinetics of strawberry (*Fragaria ananassa*): effects on antioxidant activity, anthocyanins and total phenolic content. Food Chem..

[3] Oshita D, Jardim ICSF (2014). Comparison of different sorbents in the QuEChERS method for the determination of pesticide residues in strawberries by LC–MS/MS. Chromatographia.

[4] Sun H (2021). Residue analysis and dietary exposure risk assessment of acibenzolar-S-methyl and its metabolite acibenzolar acid in potato, garlic, cabbage, grape and tomato. Ecotoxicol. Environ. Saf..

[5] Saber AN, Malhat FM, Badawy HMA, Barakat DA (2016). Dissipation dynamic, residue distribution and processing factor of hexythiazox in strawberry fruits under open field condition. Food Chem..

[6] Wang Z (2018). Screening for suitable chemical acaricides against two-spotted spider mites, *Tetranychus urticae*, on greenhouse strawberries in China. Ecotoxicol. Environ. Saf..

[7] Grimalt S, Dehouck P (2016). Review of analytical methods for the determination of pesticide residues in grapes. J. Chromatogr. A.

[8] Environmental Working Group (EWG)'s 2020 Shopper's Guide to Pesticides in Produce™. March 25, 2020. Available at https://www.ewg.org/foodnews/summary.php (accessed July 2020).

[9] Lorenz JG, Costa LLF, Suchara EA, Sant'Anna ES (2014). Multivariate optimization of the QuEChERS-GC-ECD method and pesticide investigation residues in apples, strawberries, and tomatoes produced in Brazilian south. J. Braz. Chem. Soc..

[10] Querejeta GA (2012). Environmental pesticide distribution in horticultural and floricultural periurban production units. Chemosphere.

[11] Abd-Elhaleem ZA (2020). Pesticide residues in tomato and tomato products marketed in Majmaah province, KSA, and their impact on human health. Environ. Sci. Pollut. Res..

[12] Alavanja MCR, Ross MK, Bonner MR (2013). Increased cancer burden among pesticide applicators and others due to pesticide exposure. CA Cancer J. Clin..

[13] Markel TA, Proctor C, Ying J, Winchester PD (2015). Environmental pesticides increase the risk of developing hypertrophic pyloric stenosis. J. Pediatr. Surg..

[14] Houbraken M, Bauweraerts I, Fevery D, Van Labeke MC, Spanoghe P (2016). Pesticide knowledge and practice among horticultural workers in the Lâm Đồng region, Vietnam: A case study of chrysanthemum and strawberries. Sci. Total Environ..

[15] Sharma A (2020). Global trends in pesticides: A looming threat and viable alternatives. Ecotoxicol. Environ. Saf..

[16] Commission Regulation (EC) 396/2005 of the European parliament and of the council of 23 February 2005 on maximum residue levels of pesticides in or on food and feed of plant and animal origin and amending council directive 91/414/EEC. 2005R0396-EN-13.05.2016-019.001, 1–2640.

[17] Concha-meyer A (2019). Pesticide residues quantification in frozen fruit and vegetables in Chilean domestic market using QuEChERS extraction with ultra-high-performance liquid chromatography electrospray ionization Orbitrap mass spectrometry. Food Chem..

[18] China National Standard GB 2763-2019: National food safety standard–Maximum residue limits for pesticides in food. Beijing (China): National Health Commission, Ministry of Agriculture and Rural Affairs, and State Administration for Market Regulation of the People's Republic of China.

[19] Silva RDO (2019). Efficiency of ESI and APCI ionization sources in LC-MS/MS systems for analysis of 22 pesticide residues in food matrix. Food Chem..

[20] Song NE (2019). Determination of 60 pesticides in hen eggs using the QuEChERS procedure followed by LC-MS/MS and GC-MS/MS. Food Chem..

[21] Kin CM, Huat TG (2010). Headspace solid-phase microextraction for the evaluation of pesticide residue contents in cucumber and strawberry after washing treatment. Food Chem..

[22] EU-Database. EU Pesticides Database (search pesticide residues). Available at https://ec.europa.eu/food/plant/pesticides/eu-pesticides-database/public/?event=pesticide.residue.selection&language=EN (accessed December 2020).

[23] Lozano A (2016). Miniaturisation and optimisation of the Dutch mini-Luke extraction method for implementation in the routine multi-residue analysis of pesticides in fruits and vegetables. Food Chem..

[24] Muñoz NC (2017). Determination of pesticide residues in golden berry (*Physalis peruviana* L.) by modified QuEChERS method and ultra-high performance liquid chromatography-tandem quadrupole mass spectrometry. Food Anal. Methods.

[25] Wang X, Wang S, Cai Z (2013). The latest developments and applications of mass spectrometry in food-safety and quality analysis. Trends Anal. Chem..

[26] Gaweł M (2019). Determination of neonicotinoids and 199 other pesticide residues in honey by liquid and gas chromatography coupled with tandem mass spectrometry. Food Chem..

[27] Drabova L (2019). Food fraud in oregano: pesticide residues as adulteration markers. Food Chem..

[28] Huang Y (2019). Determination of multi-pesticide residues in green tea with a modified QuEChERS protocol coupled to HPLC-MS/MS. Food Chem..

[29] Yang X, Luo J, Li S, Liu C (2016). Evaluation of nine pesticide residues in three minor tropical fruits from southern China. Food Control.

[30] Chu Y (2020). Simultaneous determination of 98 pesticide residues in strawberries using UPLC-MS/MS and GC-MS/MS. Microchem. J..

[31] Sivaperumal P, Anand P, Riddhi L (2015). Rapid determination of pesticide residues in fruits and vegetables, using ultra-high-performance liquid chromatography/time-of-flight mass spectrometry. Food Chem..

[32] Yang X, Luo J, Duan Y, Li S, Liu C (2018). Simultaneous analysis of multiple pesticide residues in minor fruits by ultrahigh-performance liquid chromatography/hybrid quadrupole time-of-fight mass spectrometry. Food Chem..

[33] Wang S, Kong C, Chen Q, Yu H (2019). Screening 89 pesticides in fishery drugs by ultrahigh performance liquid chromatography tandem quadrupole-orbitrap mass spectrometer. Molecules.

[34] Muehlwald S, Buchner N, Kroh LW (2018). Investigating the causes of low detectability of pesticides in fruits and vegetables analysed by high-performance liquid chromatography—Time-of-flight. J. Chromatogr. A.

[35] Zhang Y (2016). Rapid screening and quantification of multi-class multi-residue veterinary drugs in royal jelly by ultra performance liquid chromatography coupled to quadrupole time-of-flight mass spectrometry. Food Control.

[36] Tak V (2014). Simultaneous detection and identification of precursors, degradation and co-products of chemical warfare agents in drinking water by ultra-high performance liquid chromatography–quadrupole time-of-flight mass spectrometry. J. Chromatogr. A.

[37] Wang HX, Zhou Y, Jiang QW (2012). Simultaneous screening of estrogens, progestogens, and phenols and their metabolites in potable water and river water by ultra-performance liquid chromatography coupled with quadrupole time-of-flight mass spectrometry. Microchem. J..

[38] The Joint FAO/WHO Meeting on Pesticide Residues (JMPR). Available at http://www.fao.org/agriculture/crops/thematic-sitemap/theme/pests/lpe/lpe-c/en/ (accessed April 2021).

[39] WHO. Inventory of Evaluations Performed by the Joint Meeting on Pesticide Residues (JMPR). Available at http://apps.who.int/pesticide-residues-jmpr-database/Home/Range/All (accessed April 2021).

[40] Zhao Z (2016). Ion-exchange solid-phase extraction combined with liquid chromatography-tandem mass spectrometry for the determination of veterinary drugs in organic fertilizers. J. Chromatogr. B.

[41] SANTE. European Commission. Guidance document on analytical quality control and method validation procedures for pesticide residues analysis in food and feed. Document No. SANTE/12682/2019, 1–48.

[42] Li Z (2018). A monitoring survey and dietary risk assessment for pesticide residues on peaches in China. Regul. Toxicol. Pharmacol..

[43] Bakırcı GT, Yaman-Acay DB, Bakırcı F, Ötleş S (2014). Pesticide residues in fruits and vegetables from the Aegean region, Turkey. Food Chem..

[44] Lozowicka B (2016). Toxicological studies for adults and children of insecticide residues with common mode of action (MoA) in pome, stone, berries and other small fruit. Sci. Total Environ..

[45] Mojsak P, Łozowicka B, Kaczyński P (2018). Estimating acute and chronic exposure of children and adults to chlorpyrifos in fruit and vegetables based on the new, lower toxicology data. Ecotoxicol. Environ. Saf..

[46] Chen X, Fan X, Ma Y, Hu J (2018). Dissipation behaviour, residue distribution and dietary risk assessment of tetraconazole and kresoxim-methyl in greenhouse strawberry via RRLC-QqQ-MS/MS technique. Ecotoxicol. Environ. Saf..

[47] Heshmati A, Mehri F, Khaneghah AM (2020). Simultaneous multi-determination of pesticide residues in black tea leaves and infusion: a risk assessment study. Environ. Sci. Pollut. Res..

