# Supplementary material for: Determination and dietary risk assessment of 284 pesticide residues in local fruit cultivars in Shanghai, China
Source: Sci Rep. 2021 May 6;11:9681. doi: 10.1038/s41598-021-89204-5 (PMC8102495; doi:10.1038/s41598-021-89204-5)
Supplement: Supplementary file 1 — Supplementary Information. [file 41598_2021_89204_MOESM1_ESM.docx]

Supplementary Information

**Determination and dietary risk assessment of 284 pesticide residues in local fruit cultivars in Shanghai, China**

Yaodan Zhang ^a,b^, Wenshuai Si ^a^, Lei Chen ^a^, Guoqing Shen ^b^, Bing Bai ^a,^*, Changyan Zhou ^a,^*

^a^ Institute for Agro-food Standards and Testing Technology, Shanghai Academy of Agricultural Sciences, 1000 Jinqi Road, Shanghai 201403, China

^b^ School of Agriculture and Biology, Shanghai Jiao Tong University, 800 Dongchuan Road, Shanghai 200240, China

*Correspondence: Bing Bai, whitebing2@hotmail.com, Tel.: +86-189-1816-2357; Changyan Zhou, [zhouchangyansaas@163.com](mailto:zhouchangyansaas@163.com), Tel.: +86-189-1816-2032

**Table S1.** UPLC-QTOF/MS identification of 284 pesticides.

| **No.** | **Pesticide** | **Molecular formula** | **Retention time**  **(min)** | **Extracted mass**  **(*m/z*)** | **Found at mass**  **(*m/z*)** | **Error**  **(ppm)** | **Fragment ion 1**  **(*m/z*)** | **Fragment ion 2**  **(*m/z*)** |
| --- | --- | --- | --- | --- | --- | --- | --- | --- |
| 1 | 3-Hydroxycarbofuran | C_12_H_15_NO_4_ | 6.54 | 238.10738 [+ H] | 238.10740 | 0.1 | 163.0757 | 135.0806 |
| 2 | Abamectin B1a | C_48_H_72_O_14_ | 14.81 | 890.52603 [+ NH_4_] | 890.52735 | 1.5 | 305.2100 | 567.3305 |
| 3 | Acephate | C_4_H_10_NO_3_PS | 3.20 | 184.01918 [+ H] | 184.01920 | 0.1 | 142.9932 | 124.9822 |
| 4 | Acetamiprid | C_10_H_11_ClN_4_ | 6.60 | 223.07450 [+ H] | 223.07432 | –0.8 | 126.0102 | 90.0332 |
| 5 | Alachlor | C_14_H_20_ClNO_2_ | 11.16 | 270.12553 [+ H] | 270.12551 | –0.1 | 162.1278 | 238.1018 |
| 6 | Aldicarb-sulfoxide | C_7_H_14_N_2_O_3_S | 4.14 | 207.07979 [+ H] | 207.07971 | –0.4 | 89.0408 | 132.0467 |
| 7 | Aldoxycarb | C_7_H_14_N_2_O_4_S | 4.46 | 240.10125 [+ NH_4_] | 240.10121 | –0.2 | 86.0596 | 148.0422 |
| 8 | Ametoctradin | C_15_H_25_N_5_ | 12.45 | 276.21827 [+ H] | 276.21851 | 0.9 | 176.0919 | 149.0792 |
| 9 | Ametryn | C_9_H_17_N_5_S | 10.19 | 228.12774 [+ H] | 228.12751 | –1.0 | 186.0797 | 96.0548 |
| 10 | Amidosulfuron | C_9_H_15_N_5_O_7_S_2_ | 5.54 | 370.04857 [+ H] | 370.04885 | 0.8 | 218.0219 | 261.0278 |
| 11 | Anilofos | C_13_H_19_ClNO_3_PS_2_ | 11.72 | 368.03053 [+ H] | 368.03045 | –0.2 | 124.9811 | 170.9686 |
| 12 | Atrazine | C_8_H_14_ClN_5_ | 9.29 | 216.10105 [+ H] | 216.10107 | 0.1 | 174.0534 | 103.9998 |
| 13 | Azinphos-methyl | C_10_H_12_N_3_O_3_PS_2_ | 9.89 | 318.01305 [+ H] | 318.01314 | 0.3 | 132.0442 | 124.9825 |
| 14 | Azoxystrobin | C_22_H_17_N_3_O_5_ | 10.10 | 404.12410 [+ H] | 404.12365 | –1.1 | 372.0941 | 344.0995 |
| 15 | Benalaxyl | C_20_H_23_NO_3_ | 11.89 | 326.17507 [+ H] | 326.17516 | 0.3 | 148.1106 | 91.0528 |
| 16 | Bensulfuron-methyl | C_16_H_18_N_4_O_7_S | 8.90 | 411.09690 [+ H] | 411.09706 | 0.4 | 149.0600 | 182.0570 |
| 17 | Bifenazate | C_17_H_20_N_2_O_3_ | 10.71 | 301.15467 [+ H] | 301.15444 | –0.7 | 170.0963 | 198.0914 |
| 18 | Bifenox | C_14_H_9_Cl_2_NO_5_ | 12.21 | 341.99305 [+ H] | 341.99277 | –0.8 | 188.9487 | 309.9669 |
| 19 | Bifenthrin | C_23_H_22_ClF_3_O_2_ | 15.06 | 440.15987 [+ NH_4_] | 440.15992 | 0.1 | 181.1012 | 166.0781 |
| 20 | Bioresmethrin | C_22_H_26_O_3_ | 14.51 | 339.19547 [+ H] | 339.19486 | –1.8 | 171.0792 | 143.0848 |
| 21 | Bitertanol | C_20_H_23_N_3_O_2_ | 12.03 | 338.18630 [+ H] | 338.18663 | 1.0 | 70.0405 | 99.0805 |
| 22 | Boscalid | C_18_H_12_Cl_2_N_2_O | 10.36 | 343.03995 [+ H] | 343.03959 | –1.0 | 307.0645 | 271.0875 |
| 23 | Buprofezin | C_16_H_23_N_3_OS | 13.17 | 306.16346 [+ H] | 306.16315 | –1.0 | 106.0648 | 201.1058 |
| 24 | Butachlor | C_17_H_26_ClNO_2_ | 13.31 | 312.17248 [+ H] | 312.17228 | –0.6 | 238.0989 | 162.1278 |
| 25 | Butralin | C_14_H_21_N_3_O_4_ | 13.84 | 296.16048 [+ H] | 296.16010 | –1.3 | 240.0991 | 222.0889 |
| 26 | Cadusafos | C_10_H_23_O_2_PS_2_ | 12.43 | 271.09499 [+ H] | 271.09499 | 0.0 | 130.9382 | 158.9697 |
| 27 | Carbaryl | C_12_H_11_NO_2_ | 8.64 | 202.08626 [+ H] | 202.08640 | 0.7 | 145.0643 | 127.0544 |
| 28 | Carbendazim | C_9_H_9_N_3_O_2_ | 7.15 | 192.07675 [+ H] | 192.07660 | –0.8 | 160.0499 | 132.0555 |
| 29 | Carbofuran | C_12_H_15_NO_3_ | 8.35 | 222.11247 [+ H] | 222.11268 | 0.9 | 123.0433 | 165.0903 |
| 30 | Carboxin | C_12_H_13_NO_2_S | 8.68 | 236.07398 [+ H] | 236.07401 | 0.2 | 143.0150 | 86.9892 |
| 31 | Carfentrazone-ethyl | C_15_H_14_Cl_2_F_3_N_3_O_3_ | 11.57 | 412.04371 [+ H] | 412.04389 | 0.5 | 345.9941 | 366.0002 |
| 32 | Chlorantraniliprole | C_18_H_14_BrCl_2_N_5_O_2_ | 9.85 | 483.97579 [+ H] | 483.97536 | –0.9 | 452.9305 | 285.9193 |
| 33 | Chlorbenzuron | C_14_H_10_Cl_2_N_2_O_2_ | 11.57 | 309.01921 [+ H] | 309.01950 | 0.9 | 156.0213 | 138.9945 |
| 34 | Chlorfluazuron | C_20_H_9_Cl_3_F_5_N_3_O_3_ | 14.02 | 539.97024 [+ H] | 539.96976 | –0.9 | 382.9354 | 158.0414 |
| 35 | Chlorimuron-ethyl | C_15_H_15_ClN_4_O_6_S | 8.00 | 415.04736 [+ H] | 415.04737 | 0.0 | 186.0055 | 184.9898 |
| 36 | Chlorotoluron | C_10_H_13_ClN_2_O | 9.10 | 213.07892 [+ H] | 213.07901 | 0.4 | 72.0443 | 140.0261 |
| 37 | Chlorpyrifos-methyl | C_7_H_7_Cl_3_NO_3_PS | 12.27 | 321.90226 [+ H] | 321.90223 | –0.1 | 124.9820 | 289.8772 |
| 38 | Chlorsulfuron | C_12_H_12_ClN_5_O_4_S | 6.29 | 358.03713 [+ H] | 358.03726 | 0.4 | 141.0770 | 167.0564 |
| 39 | Cinosulfuron | C_15_H_19_N_5_O_7_S | 6.38 | 414.10780 [+ H] | 414.10820 | 1.0 | 183.0503 | 157.0711 |
| 40 | Clethodim | C_17_H_26_ClNO_3_S | 9.79 | 360.13947 [+ H] | 360.13914 | –0.9 | 164.0699 | 166.0854 |
| 41 | Clethodim-sulfone | C_17_H_26_ClNO_5_S | 6.94 | 392.12930 [+ H] | 392.12963 | 0.8 | 164.0706 | 208.1332 |
| 42 | Clethodim-sulfoxide | C_17_H_26_ClNO_4_S | 7.01 | 376.13438 [+ H] | 376.13423 | –0.4 | 206.1172 | 164.0706 |
| 43 | Clodinafop-propargyl | C_17_H_13_ClFNO_4_ | 11.46 | 350.05899 [+ H] | 350.05880 | –0.5 | 266.0368 | 238.0413 |
| 44 | Clofentezine | C_14_H_8_Cl_2_N_4_ | 12.06 | 303.01988 [+ H] | 303.01974 | –0.5 | 138.0107 | 102.0339 |
| 45 | Clomazone | C_12_H_14_ClNO_2_ | 9.87 | 240.07858 [+ H] | 240.07840 | –0.8 | 125.0140 | 89.0377 |
| 46 | Clothianidin | C_6_H_8_ClN_5_O_2_S | 6.14 | 250.01600 [+ H] | 250.01599 | 0.0 | 131.9669 | 169.0547 |
| 47 | Coumaphos | C_14_H_16_ClO_5_PS | 11.89 | 363.02174 [+ H] | 363.02161 | –0.3 | 226.9899 | 306.9560 |
| 48 | Coumoxystrobin | C_26_H_28_O_6_ | 13.29 | 437.19587 [+ H] | 437.19505 | –1.9 | 145.0630 | 205.0848 |
| 49 | Cyanazine | C_9_H_13_ClN_6_ | 8.04 | 241.09630 [+ H] | 241.09663 | 1.4 | 214.0844 | 132.0314 |
| 50 | Cyantraniliprole | C_19_H_14_BrClN_6_O_2_ | 8.90 | 475.01020 [+ H] | 475.01028 | 0.2 | 285.9204 | 443.9686 |
| 51 | Cyazofamid | C_13_H_13_ClN_4_O_2_S | 11.16 | 325.05205 [+ H] | 325.05189 | –0.5 | 108.0115 | 217.0411 |
| 52 | Cyclosulfamuron | C_17_H_19_N_5_O_6_S | 9.38 | 422.11288 [+ H] | 422.11284 | –0.1 | 218.0219 | 261.0277 |
| 53 | Cyhalofop-butyl | C_20_H_20_FNO_4_ | 12.52 | 375.17146 [+ NH_4_] | 375.17144 | –0.1 | 256.0769 | 120.0567 |
| 54 | Cyhalothrin | C_23_H_19_ClF_3_NO_3_ | 13.94 | 467.13438 [+ NH_4_] | 467.13403 | –0.8 | 255.0297 | 208.0766 |
| 55 | Cypermethrin | C_22_H_19_Cl_2_NO_3_ | 14.14 | 433.10802 [+ NH_4_] | 433.10720 | –1.9 | 191.0028 | 181.0642 |
| 56 | Cyproconazole | C_15_H_18_ClN_3_O | 10.83 | 292.12112 [+ H] | 292.12118 | 0.2 | 70.0403 | 125.0153 |
| 57 | Cyprodinil | C_14_H_15_N_3_ | 11.68 | 226.13387 [+ H] | 226.13384 | –0.1 | 210.1024 | 93.0569 |
| 58 | Cyromazine | C_6_H_10_N_6_ | 3.74 | 167.10397 [+ H] | 167.10380 | –1.1 | 68.0236 | 85.0507 |
| 59 | Deltamethrin | C_22_H_19_Br_2_NO_3_ | 14.15 | 523.00510 [+ NH_4_] | 523.00344 | –3.2 | 280.8971 | 181.0626 |
| 60 | Diafenthiuron | C_23_H_32_N_2_OS | 13.97 | 385.23081 [+ H] | 385.23074 | –0.2 | 329.1655 | 278.1501 |
| 61 | Diazinon | C_12_H_21_N_2_O_3_PS | 11.86 | 305.10833 [+ H] | 305.10836 | 0.1 | 169.0786 | 153.1015 |
| 62 | Dichlofluanid | C_9_H_11_Cl_2_FN_2_O_2_S_2_ | 10.88 | 332.96958 [+ H] | 332.96940 | –0.5 | 123.0124 | 223.9484 |
| 63 | Dichlorvos | C_4_H_7_Cl_2_O_4_P | 8.13 | 220.95318 [+ H] | 220.95302 | –0.7 | 109.0054 | 127.0150 |
| 64 | Diclofop-methyl | C_16_H_14_Cl_2_O_4_ | 12.96 | 358.06074 [+ NH_4_] | 358.06052 | –0.6 | 281.0132 | 120.0571 |
| 65 | Diethofencarb | C_14_H_21_NO_4_ | 10.12 | 268.15433 [+ H] | 268.15401 | –1.2 | 124.0385 | 152.0698 |
| 66 | Diethyl aminoethyl hexanoate | C_12_H_25_NO_2_ | 9.36 | 216.19581 [+ H] | 216.19578 | –0.1 | 143.1061 | 100.1124 |
| 67 | Difenoconazole | C_19_H_17_Cl_2_N_3_O_3_ | 12.28 | 406.07197 [+ H] | 406.07184 | –0.3 | 251.0017 | 337.0386 |
| 68 | Diflubenzuron | C_14_H_9_ClF_2_N_2_O_2_ | 11.71 | 311.03934 [+ H] | 311.03933 | 0.0 | 158.0411 | 141.0146 |
| 69 | Diflufenican | C_19_H_11_F_5_N_2_O_2_ | 12.41 | 395.08135 [+ H] | 395.08138 | 0.1 | 266.0407 | 246.0353 |
| 70 | Dimepiperate | C_15_H_21_NOS | 12.51 | 264.14166 [+ H] | 264.14184 | 0.7 | 146.0636 | 119.0861 |
| 71 | Dimethenamid-P | C_12_H_18_ClNO_2_S | 10.34 | 276.08195 [+ H] | 276.08174 | –0.8 | 244.0546 | 168.0835 |
| 72 | Dimethoate | C_5_H_12_NO_3_PS_2_ | 6.50 | 230.00690 [+ H] | 230.00670 | –0.9 | 124.9809 | 170.9688 |
| 73 | Dimethomorph | C_21_H_22_ClNO_4_ | 10.48 | 388.13101 [+ H] | 388.13063 | –1.0 | 301.0617 | 165.0543 |
| 74 | Diniconazole | C_15_H_17_Cl_2_N_3_O | 12.22 | 326.08214 [+ H] | 326.08220 | 0.2 | 70.0397 | 158.9762 |
| 75 | Dinotefuran | C_7_H_14_N_4_O_3_ | 4.21 | 203.11387 [+ H] | 203.11372 | –0.7 | 129.0892 | 114.1021 |
| 76 | Diuron | C_9_H_10_Cl_2_N_2_O | 9.51 | 233.02429 [+ H] | 233.02409 | –0.9 | 72.0441 | 159.9700 |
| 77 | Edifenphos | C_14_H_15_O_2_PS_2_ | 11.71 | 311.03239 [+ H] | 311.03235 | –0.1 | 109.0091 | 111.0249 |
| 78 | Emamectin B1a | C_49_H_75_NO_13_ | 14.28 | 886.53112 [+ H] | 886.52914 | –2.2 | 158.1150 | 302.1928 |
| 79 | Epoxiconazole | C_17_H_13_ClFN_3_O | 11.14 | 330.08039 [+ H] | 330.08024 | –0.5 | 121.0432 | 123.0227 |
| 80 | Ethametsulfuron-methyl | C_15_H_18_N_6_O_6_S | 7.17 | 411.10813 [+ H] | 411.10831 | 0.4 | 196.0823 | 168.0509 |
| 81 | Ethion | C_9_H_22_O_4_P_2_S_4_ | 13.29 | 384.99490 [+ H] | 384.99472 | –0.5 | 142.9376 | 170.9695 |
| 82 | Ethiprole | C_13_H_9_Cl_2_F_3_N_4_OS | 10.28 | 396.98990 [+ H] | 396.99014 | 0.6 | 350.9462 | 254.9683 |
| 83 | Ethirimol | C_11_H_19_N_3_O | 9.46 | 210.16009 [+ H] | 210.15998 | –0.5 | 140.1055 | 98.0586 |
| 84 | Ethoprophos | C_8_H_19_O_2_PS_2_ | 11.09 | 243.06369 [+ H] | 243.06350 | –0.8 | 130.9377 | 96.9506 |
| 85 | Ethoxyquin | C_14_H_19_NO | 11.08 | 218.15394 [+ H] | 218.15381 | –0.6 | 174.0913 | 160.0754 |
| 86 | Ethoxysulfuron | C_15_H_18_N_4_O_7_S | 8.14 | 399.09690 [+ H] | 399.09678 | –0.3 | 218.0220 | 261.0284 |
| 87 | Etofenprox | C_25_H_28_O_3_ | 14.99 | 394.23767 [+ NH_4_] | 394.23723 | –1.1 | 177.1264 | 135.0794 |
| 88 | Etoxazole | C_21_H_23_F_2_NO_2_ | 13.77 | 360.17696 [+ H] | 360.17660 | –1.0 | 141.0135 | 304.1128 |
| 89 | Famoxadone | C_22_H_18_N_2_O_4_ | 11.80 | 392.16048 [+ NH_4_] | 392.16048 | 0.0 | 239.0915 | 195.0796 |
| 90 | Fenamiphos | C_13_H_22_NO_3_PS | 11.33 | 304.11308 [+ H] | 304.11302 | –0.2 | 217.0077 | 201.9846 |
| 91 | Fenamiphos-sulfone | C_13_H_22_NO_5_PS | 8.62 | 336.10291 [+ H] | 336.10299 | 0.2 | 266.0237 | 188.0462 |
| 92 | Fenamiphos-sulfoxide | C_13_H_22_NO_4_PS | 8.47 | 320.10800 [+ H] | 320.10802 | 0.1 | 233.0027 | 171.0469 |
| 93 | Fenarimol | C_17_H_12_Cl_2_N_2_O | 10.96 | 331.03995 [+ H] | 331.04031 | 1.1 | 268.0513 | 259.0074 |
| 94 | Fenazaquin | C_20_H_22_N_2_O | 14.38 | 307.18049 [+ H] | 307.18024 | –0.8 | 57.0695 | 161.1315 |
| 95 | Fenbuconazole | C_19_H_17_ClN_4_ | 11.19 | 337.12145 [+ H] | 337.12129 | –0.5 | 125.0154 | 70.0401 |
| 96 | Fenhexamid | C_14_H_17_Cl_2_NO_2_ | 10.89 | 302.07091 [+ H] | 302.07099 | 0.3 | 97.1014 | 55.0541 |
| 97 | Fenitrothion | C_9_H_12_NO_5_PS | 10.67 | 278.02466 [+ H] | 278.02444 | –0.8 | 124.9805 | 245.9976 |
| 98 | Fenobucarb | C_12_H_17_NO_2_ | 9.99 | 208.13321 [+ H] | 208.13306 | –0.7 | 95.0492 | 77.0388 |
| 99 | Fenothiocarb | C_13_H_19_NO_2_S | 11.39 | 254.12093 [+ H] | 254.12079 | –0.5 | 72.0437 | 160.0795 |
| 100 | Fenoxanil | C_15_H_18_Cl_2_N_2_O_2_ | 11.34 | 329.08181 [+ H] | 329.08166 | –0.5 | 188.9861 | 302.0693 |
| 101 | Fenoxaprop-P-ethyl | C_18_H_16_ClNO_5_ | 12.81 | 362.07898 [+ H] | 362.07868 | –0.8 | 288.0420 | 244.0524 |
| 102 | Fenpropathrin | C_22_H_23_NO_3_ | 13.82 | 350.17507 [+ H] | 350.17487 | –0.6 | 125.0961 | 97.1024 |
| 103 | Fenpropidin | C_19_H_31_N | 11.83 | 274.25293 [+ H] | 274.25298 | 0.2 | 147.1166 | 132.0936 |
| 104 | Fenpyroximate | C_24_H_27_N_3_O_4_ | 14.02 | 422.20743 [+ H] | 422.20708 | –0.8 | 366.1410 | 135.0422 |
| 105 | Fenthion | C_10_H_15_O_3_PS_2_ | 11.65 | 279.02730 [+ H] | 279.02732 | 0.1 | 169.0129 | 247.0006 |
| 106 | Fenthion-sulfone | C_10_H_15_O_5_PS_2_ | 8.82 | 311.01713 [+ H] | 311.01738 | 0.8 | 124.9827 | 278.9925 |
| 107 | Fenthion-sulfoxide | C_10_H_15_O_4_PS_2_ | 8.61 | 295.02222 [+ H] | 295.02223 | 0.1 | 279.9989 | 109.0047 |
| 108 | Fipronil | C_12_H_4_Cl_2_F_6_N_4_OS | 11.35 | 453.97253 [+ NH_4_] | 453.97231 | –0.5 | 367.9511 | 289.9784 |
| 109 | Fipronil-sulfone | C_12_H_4_Cl_2_F_6_N_4_O_2_S | 11.76 | 469.96745 [+ NH_4_] | 469.96727 | –0.4 | 319.9842 | 243.9720 |
| 110 | Flonicamid | C_9_H_6_F_3_N_3_O | 4.96 | 230.05357 [+ H] | 230.05351 | –0.3 | 203.0403 | 148.0365 |
| 111 | Florasulam | C_12_H_8_F_3_N_5_O_3_S | 6.20 | 360.03727 [+ H] | 360.03751 | 0.6 | 129.0377 | 191.9908 |
| 112 | Fluazifop-butyl | C_19_H_20_F_3_NO_4_ | 12.93 | 384.14172 [+ H] | 384.14158 | –0.4 | 282.0713 | 328.0770 |
| 113 | Flucetosulfuron | C_18_H_22_FN_5_O_8_S | 7.05 | 488.12459 [+ H] | 488.12466 | 0.1 | 156.0751 | 273.0322 |
| 114 | Flucythrinate | C_26_H_23_F_2_NO_4_ | 13.62 | 469.19334 [+ NH_4_] | 469.19316 | –0.4 | 412.1502 | 199.0926 |
| 115 | Fludioxonil | C_12_H_6_F_2_N_2_O_2_ | 10.28 | 266.07356 [+ NH_4_] | 266.07355 | 0.0 | 229.0411 | 158.0398 |
| 116 | Flufenoxuron | C_21_H_11_ClF_6_N_2_O_3_ | 13.68 | 489.04352 [+ H] | 489.04259 | –1.9 | 158.0413 | 141.0143 |
| 117 | Flufiprole | C_16_H_10_Cl_2_F_6_N_4_OS | 11.81 | 490.99293 [+ H] | 490.99329 | 0.7 | 421.9965 | 404.9937 |
| 118 | Flumetsulam | C_12_H_9_F_2_N_5_O_2_S | 4.99 | 326.05178 [+ H] | 326.05198 | 0.6 | 129.0374 | 262.0876 |
| 119 | Flumorph | C_21_H_22_FNO_4_ | 9.65 | 372.16056 [+ H] | 372.16031 | –0.7 | 285.0924 | 165.0544 |
| 120 | Fluopicolide | C_14_H_8_Cl_3_F_3_N_2_O | 10.50 | 382.97271 [+ H] | 382.97238 | –0.9 | 172.9558 | 364.9635 |
| 121 | Fluopyram | C_16_H_11_ClF_6_N_2_O | 10.82 | 397.05369 [+ H] | 397.05387 | 0.5 | 208.0110 | 173.0187 |
| 122 | Fluoroglycofen-ethyl | C_18_H_13_ClF_3_NO_7_ | 12.56 | 465.06709 [+ NH_4_] | 465.06739 | 0.6 | 343.9900 | 300.0005 |
| 123 | Fluroxypyr | C_7_H_5_Cl_2_FN_2_O_3_ | 13.61 | 254.97340 [+ H] | 254.97294 | –1.8 | 180.9730 | 208.9688 |
| 124 | Fluroxypyr-meptyl | C_15_H_21_Cl_2_FN_2_O_3_ | 13.61 | 367.09860 [+ H] | 367.09797 | –1.7 | 208.9668 | 180.9720 |
| 125 | Flusilazole | C_16_H_15_F_2_N_3_Si | 11.29 | 316.10761 [+ H] | 316.10754 | –0.2 | 165.0688 | 247.0734 |
| 126 | Fluthiacet-methyl | C_15_H_15_ClFN_3_O_3_S_2_ | 11.57 | 404.03002 [+ H] | 404.03021 | 0.5 | 273.9952 | 344.0061 |
| 127 | Flutolanil | C_17_H_16_F_3_NO_2_ | 10.47 | 324.12059 [+ H] | 324.12039 | –0.6 | 242.0605 | 262.0669 |
| 128 | Flutriafol | C_16_H_13_F_2_N_3_O | 9.23 | 302.10995 [+ H] | 302.11027 | 1.1 | 70.0391 | 123.0235 |
| 129 | Fomesafen | C_15_H_10_ClF_3_N_2_O_6_S | 9.51 | 456.02385 [+ NH_4_] | 456.02365 | –0.4 | 343.9931 | 300.0025 |
| 130 | Fonofos | C_10_H_15_OPS_2_ | 11.75 | 247.03747 [+ H] | 247.03755 | 0.3 | 108.9875 | 80.9558 |
| 131 | Forchlorfenuron | C_12_H_10_ClN_3_O | 9.56 | 248.05852 [+ H] | 248.05851 | 0.0 | 129.0207 | 93.0445 |
| 132 | Fosthiazate | C_9_H_18_NO_3_PS_2_ | 8.99 | 284.05385 [+ H] | 284.05375 | –0.4 | 104.0160 | 227.9912 |
| 133 | Halosulfuron-methyl | C_13_H_15_ClN_6_O_7_S | 7.52 | 435.04842 [+ H] | 435.04856 | 0.3 | 182.0544 | 139.0488 |
| 134 | Haloxyfop-methyl | C_16_H_13_ClF_3_NO_4_ | 12.36 | 376.05580 [+ H] | 376.05583 | 0.1 | 316.0316 | 288.0361 |
| 135 | Hexaconazole | C_14_H_17_Cl_2_N_3_O | 11.88 | 314.08214 [+ H] | 314.08201 | –0.4 | 70.0404 | 158.9772 |
| 136 | Hexaflumuron | C_16_H_8_Cl_2_F_6_N_2_O_3_ | 12.55 | 460.98889 [+ H] | 460.98947 | 1.3 | 158.0415 | 141.0143 |
| 137 | Hexazinone | C_12_H_20_N_4_O_2_ | 8.40 | 253.16590 [+ H] | 253.16586 | –0.2 | 171.0865 | 71.0592 |
| 138 | Hexythiazox | C_17_H_21_ClN_2_O_2_S | 13.45 | 353.10850 [+ H] | 353.10828 | –0.6 | 168.0552 | 228.0215 |
| 139 | Hymexazol | C_4_H_5_NO_2_ | 1.08 | 100.03930 [+ H] | 100.03894 | –3.7 | 54.0343 | 82.0284 |
| 140 | Imazalil | C_14_H_14_Cl_2_N_2_O | 11.70 | 297.05560 [+ H] | 297.05584 | 0.8 | 158.9746 | 200.9853 |
| 141 | Imazapic | C_14_H_17_N_3_O_3_ | 3.81 | 276.13427 [+ H] | 276.13439 | 0.4 | 163.0504 | 231.1130 |
| 142 | Imazaquin | C_17_H_17_N_3_O_3_ | 6.00 | 312.13427 [+ H] | 312.13453 | 0.9 | 199.0487 | 267.1104 |
| 143 | Imazethapyr | C_15_H_19_N_3_O_3_ | 5.38 | 290.14992 [+ H] | 290.14996 | 0.1 | 245.1303 | 177.0666 |
| 144 | Imibenconazole | C_17_H_13_Cl_3_N_4_S | 13.11 | 410.99993 [+ H] | 410.99948 | –1.1 | 125.0146 | 341.9636 |
| 145 | Imidacloprid | C_9_H_10_ClN_5_O_2_ | 6.06 | 256.05958 [+ H] | 256.05972 | 0.5 | 209.0598 | 175.0984 |
| 146 | Indoxacarb | C_22_H_17_ClF_3_N_3_O_7_ | 12.37 | 528.07799 [+ H] | 528.07765 | –0.6 | 150.0098 | 218.0410 |
| 147 | Iprobenfos | C_13_H_21_O_3_PS | 11.54 | 289.10218 [+ H] | 289.10213 | –0.2 | 91.0538 | 205.0072 |
| 148 | Isazofos | C_9_H_17_ClN_3_O_3_PS | 10.80 | 314.04896 [+ H] | 314.04949 | 1.7 | 119.9951 | 162.0632 |
| 149 | Isoprocarb | C_11_H_15_NO_2_ | 9.19 | 194.11756 [+ H] | 194.11745 | –0.5 | 95.0485 | 77.0387 |
| 150 | Isoprothiolane | C_12_H_18_O_4_S_2_ | 10.54 | 291.07193 [+ H] | 291.07171 | –0.8 | 188.9669 | 144.9774 |
| 151 | Isoproturon | C_12_H_18_N_2_O | 9.39 | 207.14919 [+ H] | 207.14905 | –0.7 | 72.0443 | 165.1017 |
| 152 | Ivermectin | C_48_H_74_O_14_ | 15.54 | 892.54168 [+ NH_4_] | 892.54051 | –1.3 | 307.2279 | 551.3384 |
| 153 | Kasugamycin | C_14_H_25_N_3_O_9_ | 0.66 | 380.16636 [+ H] | 380.16698 | 1.6 | 112.0752 | 200.1037 |
| 154 | Kresoxim-methyl | C_18_H_19_NO_4_ | 11.50 | 314.13868 [+ H] | 314.13853 | –0.5 | 222.0910 | 223.0978 |
| 155 | Lactofen | C_19_H_15_ClF_3_NO_7_ | 13.03 | 479.08274 [+ NH_4_] | 479.08255 | –0.4 | 343.9915 | 300.0021 |
| 156 | Lufenuron | C_17_H_8_Cl_2_F_8_N_2_O_3_ | 13.33 | 510.98570 [+ H] | 510.98550 | –0.4 | 158.0411 | 141.0146 |
| 157 | Malathion | C_10_H_19_O_6_PS_2_ | 10.55 | 331.04335 [+ H] | 331.04357 | 0.7 | 99.0068 | 124.9814 |
| 158 | Maleic hydrazide | C_4_H_4_N_2_O_2_ | 0.68 | 113.03455 [+ H] | 113.03425 | –2.7 | 85.0382 | 67.0288 |
| 159 | Mandipropamid | C_23_H_22_ClNO_4_ | 10.37 | 412.13101 [+ H] | 412.13090 | –0.3 | 328.1097 | 125.0148 |
| 160 | Mefenacet | C_16_H_14_N_2_O_2_S | 10.76 | 299.08488 [+ H] | 299.08471 | –0.6 | 120.0790 | 148.0740 |
| 161 | Mepronil | C_17_H_19_NO_2_ | 10.61 | 270.14886 [+ H] | 270.14882 | –0.1 | 119.0486 | 228.1019 |
| 162 | Meptyldinocap-phenol | C_14_H_20_N_2_O_5_ | 12.74 | 314.17105 [+ NH_4_] | 314.17008 | –3.1 | 254.1120 | 176.1430 |
| 163 | Mesosulfuron-methyl | C_17_H_21_N_5_O_9_S_2_ | 7.12 | 504.08535 [+ H] | 504.08583 | 1.0 | 182.0564 | 306.0107 |
| 164 | Mesotrione | C_14_H_13_NO_7_S | 3.97 | 340.04855 [+ H] | 340.04865 | 0.3 | 227.9947 | 104.0124 |
| 165 | Metaflumizone | C_24_H_16_F_6_N_4_O_2_ | 13.02 | 507.12502 [+ H] | 507.12455 | –0.9 | 178.0475 | 287.0805 |
| 166 | Metalaxyl | C_15_H_21_NO_4_ | 9.51 | 280.15433 [+ H] | 280.15448 | 0.5 | 160.1113 | 192.1371 |
| 167 | Metamifop | C_23_H_18_ClFN_2_O_4_ | 12.90 | 441.10119 [+ H] | 441.10085 | –0.8 | 288.0413 | 180.0813 |
| 168 | Metamitron | C_10_H_10_N_4_O | 6.46 | 203.09274 [+ H] | 203.09259 | –0.7 | 175.0980 | 104.0497 |
| 169 | Metazachlor | C_14_H_16_ClN_3_O | 9.37 | 278.10547 [+ H] | 278.10539 | –0.3 | 134.0960 | 210.0685 |
| 170 | Methamidophos | C_2_H_8_NO_2_PS | 2.47 | 142.00862 [+ H] | 142.00855 | –0.4 | 94.0049 | 124.9822 |
| 171 | Methidathion | C_6_H_11_N_2_O_4_PS_3_ | 9.72 | 302.96914 [+ H] | 302.96904 | –0.3 | 85.0391 | 145.0069 |
| 172 | Methoxyfenozide | C_22_H_28_N_2_O_3_ | 10.69 | 369.21727 [+ H] | 369.21704 | –0.6 | 149.0588 | 133.0645 |
| 173 | Metolachlor | C_15_H_22_ClNO_2_ | 11.28 | 284.14118 [+ H] | 284.14139 | 0.7 | 252.1136 | 176.1428 |
| 174 | Metribuzin | C_8_H_14_N_4_OS | 8.30 | 215.09611 [+ H] | 215.09594 | –0.8 | 187.1018 | 84.0807 |
| 175 | Metsulfuron-methyl | C_14_H_15_N_5_O_6_S | 5.80 | 382.08158 [+ H] | 382.08173 | 0.4 | 167.0565 | 199.0055 |
| 176 | Molinate | C_9_H_17_NOS | 10.68 | 188.11036 [+ H] | 188.11016 | –1.1 | 55.0540 | 126.0906 |
| 177 | Monocrotophos | C_7_H_14_NO_5_P | 5.49 | 224.06824 [+ H] | 224.06818 | –0.3 | 127.0160 | 109.0055 |
| 178 | Myclobutanil | C_15_H_17_ClN_4_ | 10.61 | 289.12145 [+ H] | 289.12116 | –1.0 | 70.0392 | 125.0149 |
| 179 | Nicosulfuron | C_15_H_18_N_6_O_6_S | 5.38 | 411.10813 [+ H] | 411.10850 | 0.9 | 182.0553 | 213.0318 |
| 180 | Nitenpyram | C_11_H_15_ClN_4_O_2_ | 4.66 | 271.09563 [+ H] | 271.09573 | 0.4 | 126.0092 | 237.0879 |
| 181 | Novaluron | C_17_H_9_ClF_8_N_2_O_4_ | 12.67 | 493.01959 [+ H] | 493.01945 | –0.3 | 158.0402 | 141.0136 |
| 182 | Omethoate | C_5_H_12_NO_4_PS | 3.45 | 214.02974 [+ H] | 214.02963 | –0.5 | 77.0382 | 124.9814 |
| 183 | Orthosulfamuron | C_16_H_20_N_6_O_6_S | 8.25 | 425.12378 [+ H] | 425.12408 | 0.7 | 227.0493 | 199.0832 |
| 184 | Oxadiargyl | C_15_H_14_Cl_2_N_2_O_3_ | 11.97 | 341.04542 [+ H] | 341.04558 | 0.5 | 223.0037 | 151.0184 |
| 185 | Oxadiazon | C_15_H_18_Cl_2_N_2_O_3_ | 13.21 | 345.07672 [+ H] | 345.07610 | –1.8 | 219.9550 | 184.9865 |
| 186 | Oxadixyl | C_14_H_18_N_2_O_4_ | 7.83 | 279.13393 [+ H] | 279.13419 | 0.9 | 149.0234 | 132.0807 |
| 187 | Oxaziclomefone | C_20_H_19_Cl_2_NO_2_ | 12.88 | 376.08656 [+ H] | 376.08671 | 0.4 | 190.0859 | 161.0592 |
| 188 | Oxydemeton-methyl | C_6_H_15_O_4_PS_2_ | 4.98 | 247.02222 [+ H] | 247.02222 | 0.0 | 109.0032 | 169.0073 |
| 189 | Paclobutrazol | C_15_H_20_ClN_3_O | 10.45 | 294.13677 [+ H] | 294.13649 | –0.9 | 70.0394 | 125.0147 |
| 190 | Parathion | C_10_H_14_NO_5_PS | 11.43 | 292.04031 [+ H] | 292.04004 | –0.9 | 235.9772 | 123.0315 |
| 191 | Penconazole | C_13_H_15_Cl_2_N_3_ | 11.58 | 284.07158 [+ H] | 284.07190 | 1.1 | 158.9766 | 70.0395 |
| 192 | Pendimethalin | C_13_H_19_N_3_O_4_ | 13.52 | 282.14483 [+ H] | 282.14458 | –0.9 | 212.0669 | 194.0571 |
| 193 | Penoxsulam | C_16_H_14_F_5_N_5_O_5_S | 7.78 | 484.07086 [+ H] | 484.07123 | 0.8 | 195.0745 | 444.0562 |
| 194 | Permethrin | C_21_H_20_Cl_2_O_3_ | 14.84 | 408.11278 [+ NH_4_] | 408.11249 | –0.7 | 183.0805 | 155.0854 |
| 195 | Phenamacril | C_12_H_12_N_2_O_2_ | 8.29 | 217.09715 [+ H] | 217.09710 | –0.3 | 104.0498 | 171.0560 |
| 196 | Phenmedipham | C_16_H_16_N_2_O_4_ | 9.82 | 301.11828 [+ H] | 301.11830 | 0.1 | 136.0396 | 168.0659 |
| 197 | Phenthoate | C_12_H_17_O_4_PS_2_ | 11.48 | 321.03787 [+ H] | 321.03772 | –0.5 | 124.9814 | 135.0437 |
| 198 | Phorate-sulfone | C_7_H_17_O_4_PS_3_ | 9.22 | 293.00994 [+ H] | 293.01023 | 1.0 | 96.9506 | 114.9608 |
| 199 | Phorate-sulfoxide | C_7_H_17_O_3_PS_3_ | 9.08 | 277.01502 [+ H] | 277.01528 | 0.9 | 142.9374 | 96.9501 |
| 200 | Phosalone | C_12_H_15_ClNO_4_PS_2_ | 12.07 | 367.99414 [+ H] | 367.99422 | 0.2 | 182.0001 | 138.0100 |
| 201 | Phosfolan | C_7_H_14_NO_3_PS_2_ | 7.43 | 256.02255 [+ H] | 256.02244 | –0.4 | 139.9546 | 61.0101 |
| 202 | Phosfolan-methyl | C_5_H_10_NO_3_PS_2_ | 5.51 | 227.99125 [+ H] | 227.99115 | –0.5 | 167.9868 | 109.0038 |
| 203 | Phosmet | C_11_H_12_NO_4_PS_2_ | 9.91 | 318.00182 [+ H] | 318.00202 | 0.6 | 160.0391 | 133.0279 |
| 204 | Phosphamidon | C_10_H_19_ClNO_5_P | 7.87 | 300.07622 [+ H] | 300.07649 | 0.9 | 127.0158 | 174.0689 |
| 205 | Phoxim | C_12_H_15_N_2_O_3_PS | 11.94 | 299.06138 [+ H] | 299.06158 | 0.7 | 77.0387 | 129.0448 |
| 206 | Picoxystrobin | C_18_H_16_F_3_NO_4_ | 11.34 | 368.11042 [+ H] | 368.11043 | 0.0 | 145.0644 | 205.0865 |
| 207 | Pinoxaden | C_23_H_32_N_2_O_4_ | 12.15 | 401.24348 [+ H] | 401.24342 | –0.2 | 317.1836 | 57.0694 |
| 208 | Piperonyl-butoxide | C_19_H_30_O_5_ | 13.28 | 356.24315 [+ NH_4_] | 356.24316 | 0.0 | 177.0883 | 119.0837 |
| 209 | Pirimicarb | C_11_H_18_N_4_O_2_ | 9.17 | 239.15025 [+ H] | 239.15047 | 0.9 | 72.0432 | 182.1275 |
| 210 | Pirimiphos-methyl | C_11_H_20_N_3_O_3_PS | 12.15 | 306.10358 [+ H] | 306.10334 | –0.8 | 164.1162 | 108.0539 |
| 211 | Pretilachlor | C_17_H_26_ClNO_2_ | 12.74 | 312.17248 [+ H] | 312.17257 | 0.3 | 252.1148 | 176.1436 |
| 212 | Prochloraz | C_15_H_16_Cl_3_N_3_O_2_ | 12.06 | 376.03809 [+ H] | 376.03774 | –0.9 | 308.0016 | 265.9550 |
| 213 | Profenofos | C_11_H_15_BrClO_3_PS | 12.81 | 374.94017 [+ H] | 374.93953 | –1.7 | 304.8599 | 286.8495 |
| 214 | Prometryn | C_10_H_19_N_5_S | 11.00 | 242.14339 [+ H] | 242.14336 | –0.2 | 158.0485 | 200.0953 |
| 215 | Propachlor | C_11_H_14_ClNO | 9.41 | 212.08367 [+ H] | 212.08350 | –0.8 | 170.0363 | 94.0648 |
| 216 | Propamocarb | C_9_H_20_N_2_O_2_ | 4.97 | 189.15975 [+ H] | 189.15966 | –0.5 | 102.0551 | 74.0236 |
| 217 | Propanil | C_9_H_9_Cl_2_NO | 10.17 | 218.01340 [+ H] | 218.01309 | –1.4 | 127.0184 | 161.9871 |
| 218 | Propargite | C_19_H_26_O_4_S | 13.71 | 368.18901 [+ NH_4_] | 368.18854 | –1.3 | 175.1103 | 231.1729 |
| 219 | Propiconazole | C_15_H_17_Cl_2_N_3_O_2_ | 11.80 | 342.07706 [+ H] | 342.07712 | 0.2 | 158.9759 | 69.0706 |
| 220 | Propyzamide | C_12_H_11_Cl_2_NO | 10.56 | 256.02905 [+ H] | 256.02873 | –1.2 | 172.9566 | 189.9821 |
| 221 | Prothioconazole-desthiometabolite | C_14_H_15_Cl_2_N_3_O | 11.20 | 312.06649 [+ H] | 312.06630 | –0.6 | 70.0393 | 125.0143 |
| 222 | Pymetrozine | C_10_H_11_N_5_O | 5.39 | 218.10364 [+ H] | 218.10355 | –0.4 | 105.0438 | 79.0410 |
| 223 | Pyraclostrobin | C_19_H_18_ClN_3_O_4_ | 11.97 | 388.10586 [+ H] | 388.10604 | 0.5 | 163.0614 | 194.0795 |
| 224 | Pyraflufen-ethyl | C_15_H_13_Cl_2_F_3_N_2_O_4_ | 11.72 | 413.02772 [+ H] | 413.02758 | –0.3 | 338.9887 | 288.9929 |
| 225 | Pyrametostrobin | C_21_H_23_N_3_O_4_ | 11.52 | 382.17613 [+ H] | 382.17642 | 0.8 | 163.0616 | 149.0461 |
| 226 | Pyraoxystrobin | C_22_H_21_ClN_2_O_4_ | 12.02 | 413.12626 [+ H] | 413.12633 | 0.2 | 145.0644 | 205.0853 |
| 227 | Pyrazosulfuron-ethyl | C_14_H_18_N_6_O_7_S | 7.39 | 415.10305 [+ H] | 415.10299 | –0.1 | 182.0548 | 139.0490 |
| 228 | Pyrethrin I | C_21_H_28_O_3_ | 13.87 | 329.21112 [+ H] | 329.21111 | 0.0 | 161.0950 | 143.0847 |
| 229 | Pyrethrin II | C_22_H_28_O_5_ | 12.55 | 373.20095 [+ H] | 373.20112 | 0.4 | 161.0961 | 143.0841 |
| 230 | Pyridaben | C_19_H_25_ClN_2_OS | 14.35 | 365.14489 [+ H] | 365.14450 | –1.1 | 147.1145 | 309.0786 |
| 231 | Pyriftalid | C_15_H_14_N_2_O_4_S | 9.98 | 319.07471 [+ H] | 319.07494 | 0.7 | 139.0497 | 179.0156 |
| 232 | Pyrimethanil | C_12_H_13_N_3_ | 10.21 | 200.11822 [+ H] | 200.11810 | –0.6 | 183.0919 | 182.0841 |
| 233 | Pyriproxyfen | C_20_H_19_NO_3_ | 13.25 | 322.14377 [+ H] | 322.14352 | –0.8 | 96.0437 | 185.0596 |
| 234 | Quinalphos | C_12_H_15_N_2_O_3_PS | 11.52 | 299.06138 [+ H] | 299.06151 | 0.5 | 163.0320 | 147.0547 |
| 235 | Quinoxyfen | C_15_H_8_Cl_2_FNO | 13.40 | 308.00397 [+ H] | 308.00359 | –1.2 | 196.9789 | 272.0269 |
| 236 | Quizalofop-ethyl | C_19_H_17_ClN_2_O_4_ | 12.86 | 373.09496 [+ H] | 373.09512 | 0.4 | 299.0578 | 271.0627 |
| 237 | Rimsulfuron | C_14_H_17_N_5_O_7_S_2_ | 5.90 | 432.06422 [+ H] | 432.06445 | 0.5 | 182.0560 | 325.0957 |
| 238 | Rotenone | C_23_H_22_O_6_ | 11.30 | 395.14892 [+ H] | 395.14889 | –0.1 | 213.0906 | 192.0779 |
| 239 | Saflufenacil | C_17_H_17_ClF_4_N_4_O_5_S | 8.76 | 501.06171 [+ H] | 501.06185 | 0.3 | 348.9987 | 366.0261 |
| 240 | Sethoxydim | C_17_H_29_NO_3_S | 10.43 | 328.19409 [+ H] | 328.19370 | –1.2 | 178.0850 | 180.1004 |
| 241 | Simazine | C_7_H_12_ClN_5_ | 8.35 | 202.08540 [+ H] | 202.08534 | –0.3 | 132.0331 | 124.0875 |
| 242 | Simetryn | C_8_H_15_N_5_S | 9.31 | 214.11209 [+ H] | 214.11200 | –0.4 | 124.0864 | 144.0587 |
| 243 | Spinetoram A | C_42_H_69_NO_10_ | 15.48 | 748.49942 [+ H] | 748.49806 | –1.8 | 142.1222 | 203.1286 |
| 244 | Spinosad A | C_41_H_65_NO_10_ | 15.16 | 732.46812 [+ H] | 732.46743 | –0.9 | 142.1218 | 184.0729 |
| 245 | Spinosad D | C_42_H_67_NO_10_ | 15.44 | 746.48377 [+ H] | 746.48259 | –1.6 | 142.1223 | 189.1126 |
| 246 | Spirodiclofen | C_21_H_24_Cl_2_O_4_ | 14.12 | 411.11244 [+ H] | 411.11180 | –1.6 | 313.0389 | 71.0857 |
| 247 | Spirotetramat | C_21_H_27_NO_5_ | 10.99 | 374.19620 [+ H] | 374.19630 | 0.3 | 216.0999 | 270.1465 |
| 248 | Spirotetramat-enol | C_18_H_23_NO_3_ | 7.11 | 302.17507 [+ H] | 302.17537 | 1.0 | 216.1017 | 270.1494 |
| 249 | Sulcotrione | C_14_H_13_ClO_5_S | 4.63 | 329.02450 [+ H] | 329.02463 | 0.4 | 139.0383 | 111.0436 |
| 250 | Sulfotep | C_8_H_20_O_5_P_2_S_2_ | 11.60 | 323.03002 [+ H] | 323.03025 | 0.7 | 114.9611 | 142.9925 |
| 251 | Sulfoxaflor | C_10_H_10_F_3_N_3_OS | 6.71 | 278.05695 [+ H] | 278.05727 | 1.2 | 174.0525 | 154.0461 |
| 252 | tau-Fluvalinate | C_26_H_22_ClF_3_N_2_O_3_ | 14.53 | 503.13438 [+ H] | 503.13410 | –0.6 | 181.0643 | 208.0754 |
| 253 | Tebuconazole | C_16_H_22_ClN_3_O | 11.65 | 308.15242 [+ H] | 308.15267 | 0.8 | 70.0398 | 125.0146 |
| 254 | Tebufenozide | C_22_H_28_N_2_O_2_ | 11.42 | 353.22235 [+ H] | 353.22215 | –0.6 | 133.0630 | 105.0678 |
| 255 | Teflubenzuron | C_14_H_6_Cl_2_F_4_N_2_O_2_ | 13.12 | 380.98152 [+ H] | 380.98132 | –0.5 | 158.0410 | 141.0145 |
| 256 | Terbufos-sulfone | C_9_H_21_O_4_PS_3_ | 10.07 | 338.06779 [+ NH_4_] | 338.06741 | –1.1 | 114.9611 | 171.0241 |
| 257 | Terbufos-sulfoxide | C_9_H_21_O_3_PS_3_ | 10.10 | 305.04633 [+ H] | 305.04600 | –1.1 | 130.9372 | 187.0001 |
| 258 | Terbuthylazine | C_9_H_16_ClN_5_ | 10.35 | 230.11670 [+ H] | 230.11654 | –0.7 | 174.0536 | 132.0319 |
| 259 | Thiabendazole | C_10_H_7_N_3_S | 7.90 | 202.04335 [+ H] | 202.04330 | –0.2 | 175.0313 | 131.0593 |
| 260 | Thiacloprid | C_10_H_9_ClN_4_S | 7.13 | 253.03092 [+ H] | 253.03108 | 0.6 | 126.0089 | 98.9981 |
| 261 | Thiamethoxam | C_8_H_10_ClN_5_O_3_S | 5.19 | 292.02657 [+ H] | 292.02673 | 0.6 | 181.0548 | 211.0647 |
| 262 | Thidiazuron | C_9_H_8_N_4_OS | 8.38 | 221.04916 [+ H] | 221.04899 | –0.7 | 102.0123 | 127.9916 |
| 263 | Thifensulfuron-methyl | C_12_H_13_N_5_O_6_S_2_ | 5.81 | 388.03800 [+ H] | 388.03834 | 0.9 | 167.0562 | 204.9618 |
| 264 | Thifluzamide | C_13_H_6_Br_2_F_6_N_2_O_2_S | 11.12 | 528.84733 [+ H] | 528.84819 | 1.6 | 488.8340 | 148.0019 |
| 265 | Thiobencarb | C_12_H_16_ClNOS | 12.10 | 258.07139 [+ H] | 258.07128 | –0.4 | 125.0147 | 89.0387 |
| 266 | Thiodicarb | C_10_H_18_N_4_O_4_S_3_ | 9.04 | 355.05630 [+ H] | 355.05591 | –1.1 | 88.0216 | 107.9939 |
| 267 | Thiophanate-methyl | C_12_H_14_N_4_O_4_S_2_ | 8.30 | 343.05293 [+ H] | 343.05327 | 1.0 | 151.0306 | 160.0491 |
| 268 | Tolclofos-methyl | C_9_H_11_Cl_2_O_3_PS | 12.04 | 300.96164 [+ H] | 300.96183 | 0.6 | 174.9711 | 268.9358 |
| 269 | Tolfenpyrad | C_21_H_22_ClN_3_O_2_ | 13.13 | 384.14733 [+ H] | 384.14666 | –1.7 | 197.0969 | 171.0328 |
| 270 | Tolylfluanid | C_10_H_13_Cl_2_FN_2_O_2_S_2_ | 11.59 | 346.98523 [+ H] | 346.98550 | 0.8 | 137.0290 | 237.9653 |
| 271 | Triadimefon | C_14_H_16_ClN_3_O_2_ | 10.64 | 294.10038 [+ H] | 294.10025 | –0.5 | 197.0734 | 69.0701 |
| 272 | Triadimenol | C_14_H_18_ClN_3_O_2_ | 10.79 | 296.11603 [+ H] | 296.11610 | 0.2 | 70.0404 | 227.0856 |
| 273 | Triasulfuron | C_14_H_16_ClN_5_O_5_S | 6.82 | 402.06334 [+ H] | 402.06331 | –0.1 | 167.0566 | 141.0772 |
| 274 | Triazophos | C_12_H_16_N_3_O_3_PS | 10.78 | 314.07228 [+ H] | 314.07171 | –1.8 | 162.0647 | 119.0597 |
| 275 | Tribenuron-methyl | C_15_H_17_N_5_O_6_S | 6.89 | 396.09723 [+ H] | 396.09763 | 1.0 | 155.0921 | 181.0712 |
| 276 | Trichlorfon | C_4_H_8_Cl_3_O_4_P | 6.41 | 256.92986 [+ H] | 256.93001 | 0.6 | 109.0043 | 78.9933 |
| 277 | Tricyclazole | C_9_H_7_N_3_S | 7.46 | 190.04335 [+ H] | 190.04323 | –0.6 | 163.0324 | 136.0213 |
| 278 | Trifloxystrobin | C_20_H_19_F_3_N_2_O_4_ | 12.46 | 409.13697 [+ H] | 409.13737 | 1.0 | 186.0519 | 206.0809 |
| 279 | Triflumizole | C_15_H_15_ClF_3_N_3_O | 12.60 | 346.09285 [+ H] | 346.09297 | 0.3 | 278.0568 | 73.0646 |
| 280 | Triflumuron | C_15_H_10_ClF_3_N_2_O_3_ | 11.99 | 359.04048 [+ H] | 359.04061 | 0.4 | 156.0207 | 138.9944 |
| 281 | Trinexapac-ethyl | C_13_H_16_O_5_ | 6.09 | 253.10705 [+ H] | 253.10715 | 0.4 | 69.0334 | 165.0180 |
| 282 | Uniconazole | C_15_H_18_ClN_3_O | 11.24 | 292.12112 [+ H] | 292.12118 | 0.2 | 70.0405 | 125.0158 |
| 283 | Vamidothion | C_8_H_18_NO_4_PS_2_ | 6.54 | 288.04877 [+ H] | 288.04883 | 0.2 | 146.0627 | 118.0315 |
| 284 | Zoxamide | C_14_H_16_Cl_3_NO_2_ | 11.87 | 336.03194 [+ H] | 336.03218 | 0.7 | 186.9706 | 158.9756 |

**Table S2.** Analytical parameters for the analysis of pesticides in strawberry matrix by UPLC-QTOF/MS method.

| **No.** | **Pesticide** | **Linearity (*r^2^*)** | **Equation** | **LOD^a^ (µg/kg)** | **LOQ^b^ (µg/kg)** | **Recovery (%)** | | **RSD^c^ (%)** | |
| --- | --- | --- | --- | --- | --- | --- | --- | --- | --- |
|  |  |  |  |  |  | **10 µg/kg** | **100 µg/kg** | **10 µg/kg** | **100 µg/kg** |
| 1 | 3-Hydroxycarbofuran | 0.9993 | y = 1529.06247 x – 2565.53895 | 3.0 | 10.0 | 97 | 86 | 1.5 | 3.2 |
| 2 | Abamectin B1a | 0.9969 | y = 510.95769 x – 670.72062 | 5.0 | 15.0 | 118 | 113 | 15.5 | 13.4 |
| 3 | Acephate | 0.9988 | y = 235.91636 x – 130.19638 | 2.0 | 6.0 | 75 | 89 | 2.5 | 4.1 |
| 4 | Acetamiprid | 0.9988 | y = 6575.13567 x + 5371.59622 | 1.0 | 3.0 | 115 | 104 | 3.5 | 2.3 |
| 5 | Alachlor | 0.9994 | y = 2486.24886 x + 501.75261 | 2.0 | 6.0 | 100 | 90 | 3.3 | 2.0 |
| 6 | Aldicarb-sulfoxide | 0.9988 | y = 642.30833 x – 248.87903 | 3.0 | 10.0 | 75 | 85 | 5.6 | 6.3 |
| 7 | Aldoxycarb | 0.9984 | y = 940.13211 x – 779.23853 | 2.0 | 6.0 | 109 | 97 | 1.8 | 2.7 |
| 8 | Ametoctradin | 0.9988 | y = 2670.87873 x + 15355.00860 | 3.0 | 10.0 | 111 | 104 | 1.3 | 3.3 |
| 9 | Ametryn | 0.9988 | y = 14830.98973 x + 1228.94955 | 0.6 | 2.0 | 116 | 88 | 4.8 | 2.1 |
| 10 | Amidosulfuron | 0.9991 | y = 1480.92170 x + 1032.80541 | 1.0 | 3.0 | 120 | 109 | 3.2 | 2.9 |
| 11 | Anilofos | 0.9980 | y = 7726.60234 x + 9720.21893 | 1.0 | 3.0 | 106 | 82 | 1.2 | 0.9 |
| 12 | Atrazine | 0.9982 | y = 7561.38204 x + 936.83876 | 1.0 | 3.0 | 73 | 86 | 2.2 | 2.6 |
| 13 | Azinphos-methyl | 0.9973 | y = 1038.67337 x – 1389.94354 | 3.0 | 10.0 | 90 | 86 | 7.4 | 5.1 |
| 14 | Azoxystrobin | 0.9987 | y = 12816.80318 x – 1065.47452 | 0.6 | 2.0 | 117 | 106 | 1.6 | 2.5 |
| 15 | Benalaxyl | 0.9986 | y = 11506.09797 x + 9598.80218 | 1.0 | 3.0 | 114 | 98 | 1.1 | 2.0 |
| 16 | Bensulfuron-methyl | 0.9981 | y = 3123.10710 x + 5247.15709 | 1.0 | 3.0 | 129 | 112 | 3.3 | 2.8 |
| 17 | Bifenazate | 0.9946 | y = 1977.86167 x + 8429.15517 | 10.0 | 30.0 | 79 | 95 | 3.2 | 7.9 |
| 18 | Bifenox | 0.9994 | y = 332.54756 x + 594.47903 | 3.0 | 10.0 | 106 | 92 | 6.8 | 5.9 |
| 19 | Bifenthrin | 0.9994 | y = 2358.35764 x – 1437.12985 | 2.0 | 6.0 | 123 | 119 | 14.2 | 13.8 |
| 20 | Bioresmethrin | 0.9982 | y = 6636.13108 x – 1814.32348 | 1.0 | 3.0 | 72 | 99 | 4.6 | 1.0 |
| 21 | Bitertanol | 0.9990 | y = 1382.29578 x + 653.77380 | 3.0 | 10.0 | 111 | 107 | 5.1 | 4.9 |
| 22 | Boscalid | 0.9984 | y = 2288.53188 x + 2235.64084 | 0.6 | 2.0 | 112 | 105 | 0.2 | 3.2 |
| 23 | Buprofezin | 0.9985 | y = 13896.13765 x – 4824.53140 | 0.6 | 2.0 | 111 | 93 | 1.5 | 2.3 |
| 24 | Butachlor | 0.9989 | y = 2327.74774 x + 2460.22179 | 2.0 | 6.0 | 102 | 90 | 6.1 | 5.4 |
| 25 | Butralin | 0.9987 | y = 2246.56985 x + 355.14411 | 2.0 | 6.0 | 84 | 93 | 6.4 | 7.1 |
| 26 | Cadusafos | 0.9985 | y = 5456.44948 x + 4320.42991 | 1.0 | 3.0 | 116 | 104 | 3.9 | 0.8 |
| 27 | Carbaryl | 0.9982 | y = 389.42825 x + 1283.94533 | 10.0 | 30.0 | 90 | 89 | 7.4 | 9.3 |
| 28 | Carbendazim | 0.9986 | y = 7199.51606 x + 14080.85551 | 1.0 | 3.0 | 106 | 108 | 4.8 | 2.9 |
| 29 | Carbofuran | 0.9995 | y = 5201.41828 x + 389.75073 | 2.0 | 6.0 | 101 | 93 | 2.1 | 3.8 |
| 30 | Carboxin | 0.9992 | y = 8136.51452 x + 8509.41007 | 2.0 | 6.0 | 113 | 99 | 4.6 | 3.2 |
| 31 | Carfentrazone-ethyl | 0.9995 | y = 843.84804 x – 497.54764 | 2.0 | 6.0 | 119 | 107 | 3.2 | 2.9 |
| 32 | Chlorantraniliprole | 0.9988 | y = 1697.10464 x – 94.14182 | 0.6 | 2.0 | 109 | 104 | 4.2 | 2.1 |
| 33 | Chlorbenzuron | 0.9982 | y = 1045.46772 x + 2328.62422 | 2.0 | 6.0 | 109 | 101 | 1.4 | 2.4 |
| 34 | Chlorfluazuron | 0.9990 | y = 872.09373 x – 544.48043 | 3.0 | 10.0 | 89 | 79 | 5.5 | 4.9 |
| 35 | Chlorimuron-ethyl | 0.9988 | y = 1848.65294 x + 1613.67519 | 1.0 | 3.0 | 116 | 111 | 6.3 | 5.2 |
| 36 | Chlorotoluron | 0.9994 | y = 6361.82914 x + 9584.34881 | 1.0 | 3.0 | 110 | 103 | 3.2 | 3.0 |
| 37 | Chlorpyrifos-methyl | 0.9989 | y = 702.27876 x – 351.34212 | 1.0 | 3.0 | 107 | 112 | 3.6 | 4.8 |
| 38 | Chlorsulfuron | 0.9986 | y = 1417.54365 x – 558.32768 | 1.0 | 3.0 | 125 | 121 | 5.0 | 2.8 |
| 39 | Cinosulfuron | 0.9986 | y = 3551.91130 x + 104.10909 | 0.6 | 2.0 | 118 | 104 | 4.3 | 2.9 |
| 40 | Clethodim | 0.9934 | y = 3393.55643 x + 5074.05060 | 1.0 | 3.0 | 113 | 102 | 3.5 | 2.2 |
| 41 | Clethodim-sulfone | 0.9952 | y = 1377.39764 x + 8800.00994 | 2.0 | 6.0 | 117 | 106 | 5.4 | 4.0 |
| 42 | Clethodim-sulfoxide | 0.9965 | y = 2011.64806 x + 11948.3928 | 2.0 | 6.0 | 101 | 103 | 6.0 | 5.7 |
| 43 | Clodinafop-propargyl | 0.9980 | y = 5781.93288 x – 289.82815 | 1.0 | 3.0 | 105 | 100 | 3.3 | 2.2 |
| 44 | Clofentezine | 0.9983 | y = 1298.51113 x + 960.26622 | 2.0 | 6.0 | 98 | 91 | 5.3 | 4.9 |
| 45 | Clomazone | 0.9995 | y = 3498.01411 x + 276.40801 | 0.6 | 2.0 | 115 | 108 | 2.4 | 1.3 |
| 46 | Clothianidin | 0.9986 | y = 1174.33455 x + 631.08474 | 0.6 | 2.0 | 82 | 101 | 6.0 | 7.3 |
| 47 | Coumaphos | 0.9987 | y = 7034.66220 x + 288.76278 | 1.0 | 3.0 | 111 | 95 | 2.9 | 1.6 |
| 48 | Coumoxystrobin | 0.9980 | y = 395.94573 x + 1097.52784 | 5.0 | 15.0 | 116 | 97 | 8.2 | 6.9 |
| 49 | Cyanazine | 0.9975 | y = 4361.01101 x + 3108.51751 | 1.0 | 3.0 | 113 | 99 | 1.7 | 2.3 |
| 50 | Cyantraniliprole | 0.9957 | y = 1047.82834 x – 1877.37963 | 3.0 | 10.0 | 114 | 96 | 5.6 | 4.7 |
| 51 | Cyazofamid | 0.9989 | y = 2848.01535 x + 4256.41639 | 1.0 | 3.0 | 101 | 108 | 3.4 | 0.4 |
| 52 | Cyclosulfamuron | 0.9990 | y = 4165.97290 x + 1049.73919 | 0.6 | 2.0 | 118 | 109 | 4.2 | 2.0 |
| 53 | Cyhalofop-butyl | 0.9990 | y = 954.53095 x + 1962.37484 | 3.0 | 10.0 | 106 | 93 | 1.1 | 3.1 |
| 54 | Cyhalothrin | 0.9979 | y = 3135.69997 x – 1225.14851 | 2.0 | 6.0 | 79 | 92 | 4.4 | 5.1 |
| 55 | Cypermethrin | 0.9995 | y = 2095.53298 x – 1561.46272 | 3.0 | 10.0 | 80 | 96 | 6.6 | 7.9 |
| 56 | Cyproconazole | 0.9984 | y = 1984.61010 x + 1904.62788 | 1.0 | 3.0 | 100 | 84 | 3.4 | 2.9 |
| 57 | Cyprodinil | 0.9993 | y = 9011.51060 x + 7112.83724 | 1.0 | 3.0 | 110 | 89 | 1.7 | 1.3 |
| 58 | Cyromazine | 0.9989 | y = 2486.36352 x + 1892.10864 | 2.0 | 6.0 | 53 | 64 | 4.2 | 14.4 |
| 59 | Deltamethrin | 0.9989 | y = 1852.91964 x – 274.15203 | 3.0 | 10.0 | 88 | 82 | 4.7 | 3.5 |
| 60 | Diafenthiuron | 0.9955 | y = 4116.81131 x – 11226.82139 | 5.0 | 15.0 | 69 | 72 | 1.5 | 11.6 |
| 61 | Diazinon | 0.9995 | y = 8085.50706 x – 4563.47400 | 1.0 | 3.0 | 109 | 105 | 3.4 | 1.3 |
| 62 | Dichlofluanid | 0.9953 | y = 761.74849 x – 96.40865 | 2.0 | 6.0 | 97 | 106 | 9.7 | 7.8 |
| 63 | Dichlorvos | 0.9981 | y = 834.30072 x + 1435.81764 | 2.0 | 6.0 | 99 | 112 | 4.3 | 4.8 |
| 64 | Diclofop-methyl | 0.9984 | y = 1600.11557 x + 678.32822 | 2.0 | 6.0 | 104 | 90 | 3.0 | 2.6 |
| 65 | Diethofencarb | 0.9993 | y = 2480.35475 x + 11188.83759 | 3.0 | 10.0 | 114 | 101 | 3.7 | 3.3 |
| 66 | Diethyl aminoethyl hexanoate | 0.9953 | y = 2948.05757 x – 8543.51406 | 5.0 | 15.0 | 119 | 105 | 4.3 | 3.8 |
| 67 | Difenoconazole | 0.9994 | y = 3277.43620 x + 805.66880 | 0.6 | 2.0 | 115 | 95 | 3.1 | 2.6 |
| 68 | Diflubenzuron | 0.9995 | y = 1131.40771 x + 891.63259 | 2.0 | 6.0 | 94 | 106 | 3.3 | 2.3 |
| 69 | Diflufenican | 0.9989 | y = 2312.86813 x + 1619.03439 | 1.0 | 3.0 | 111 | 98 | 3.9 | 2.6 |
| 70 | Dimepiperate | 0.9985 | y = 1181.21072 x + 99.15832 | 3.0 | 10.0 | 73 | 83 | 7.5 | 8.4 |
| 71 | Dimethenamid-P | 0.9991 | y = 4266.68967 x – 525.40770 | 0.6 | 2.0 | 111 | 112 | 2.6 | 1.7 |
| 72 | Dimethoate | 0.9983 | y = 2691.29484 x + 854.64865 | 0.6 | 2.0 | 113 | 89 | 2.7 | 1.4 |
| 73 | Dimethomorph | 0.9988 | y = 3561.81603 x + 1079.83712 | 1.0 | 3.0 | 119 | 103 | 0.7 | 2.6 |
| 74 | Diniconazole | 0.9990 | y = 2060.68585 x + 374.32403 | 0.6 | 2.0 | 112 | 100 | 1.5 | 2.3 |
| 75 | Dinotefuran | 0.9993 | y = 1755.68323 x + 323.59509 | 1.0 | 3.0 | 113 | 98 | 6.0 | 5.2 |
| 76 | Diuron | 0.9978 | y = 4055.86915 x + 1619.72624 | 0.6 | 2.0 | 111 | 106 | 0.9 | 2.8 |
| 77 | Edifenphos | 0.9990 | y = 8645.25755 x – 4068.00566 | 2.0 | 6.0 | 110 | 83 | 2.0 | 1.5 |
| 78 | Emamectin B1a | 0.9972 | y = 6766.34918 x – 3166.12496 | 1.0 | 3.0 | 92 | 97 | 3.6 | 4.8 |
| 79 | Epoxiconazole | 0.9995 | y = 4430.22895 x + 1381.40098 | 0.6 | 2.0 | 96 | 102 | 4.7 | 5.0 |
| 80 | Ethametsulfuron-methyl | 0.9987 | y = 3629.36090 x – 115.55971 | 0.6 | 2.0 | 137 | 122 | 5.3 | 4.7 |
| 81 | Ethion | 0.9986 | y = 5754.61757 x – 978.42955 | 0.6 | 2.0 | 108 | 91 | 3.2 | 2.8 |
| 82 | Ethiprole | 0.9982 | y = 1898.09710 x + 3286.25643 | 1.0 | 3.0 | 110 | 105 | 6.1 | 5.8 |
| 83 | Ethirimol | 0.9989 | y = 7820.55874 x + 2968.88454 | 0.6 | 2.0 | 85 | 105 | 2.2 | 2.8 |
| 84 | Ethoprophos | 0.9997 | y = 6527.17926 x + 3128.78474 | 1.0 | 3.0 | 99 | 106 | 0.6 | 1.7 |
| 85 | Ethoxyquin | 0.9996 | y = 2960.32151 x – 1442.19852 | 3.0 | 10.0 | 116 | 120 | 0.2 | 3.2 |
| 86 | Ethoxysulfuron | 0.9981 | y = 2544.79896 x – 352.49050 | 0.6 | 2.0 | 130 | 125 | 1.3 | 2.1 |
| 87 | Etofenprox | 0.9987 | y = 7985.43993 x + 8698.18799 | 1.0 | 3.0 | 107 | 95 | 1.9 | 10.8 |
| 88 | Etoxazole | 0.9991 | y = 20836.14427 x – 2786.42032 | 0.6 | 2.0 | 77 | 94 | 3.4 | 1.6 |
| 89 | Famoxadone | 0.9986 | y = 658.84323 x – 2383.79597 | 6.0 | 20.0 | 103 | 93 | 2.4 | 3.4 |
| 90 | Fenamiphos | 0.9987 | y = 9583.49927 x + 12410.86132 | 1.0 | 3.0 | 109 | 106 | 2.7 | 3.6 |
| 91 | Fenamiphos-sulfone | 0.9992 | y = 10134.40645 x – 1553.86967 | 0.6 | 2.0 | 119 | 107 | 1.3 | 2.5 |
| 92 | Fenamiphos-sulfoxide | 0.9959 | y = 9641.76646 x + 16703.91399 | 1.0 | 3.0 | 79 | 87 | 1.0 | 2.9 |
| 93 | Fenarimol | 0.9993 | y = 1560.88928 x – 263.21950 | 2.0 | 6.0 | 104 | 114 | 1.4 | 3.4 |
| 94 | Fenazaquin | 0.9993 | y = 15402.07954 x + 2287.26249 | 1.0 | 3.0 | 109 | 107 | 3.7 | 8.6 |
| 95 | Fenbuconazole | 0.9994 | y = 2960.97570 x + 2083.74238 | 2.0 | 6.0 | 89 | 98 | 8.0 | 6.0 |
| 96 | Fenhexamid | 0.9987 | y = 1977.90241 x + 429.13648 | 2.0 | 6.0 | 95 | 106 | 0.6 | 2.6 |
| 97 | Fenitrothion | 0.9970 | y = 630.75052 x – 68.84156 | 2.0 | 6.0 | 84 | 91 | 9.8 | 8.1 |
| 98 | Fenobucarb | 0.9987 | y = 1784.55944 x + 3245.06223 | 3.0 | 10.0 | 119 | 111 | 1.5 | 0.9 |
| 99 | Fenothiocarb | 0.9994 | y = 4722.51693 x + 6348.74235 | 1.0 | 3.0 | 111 | 97 | 1.9 | 2.6 |
| 100 | Fenoxanil | 0.9969 | y = 3041.73964 x + 4998.27397 | 2.0 | 6.0 | 102 | 99 | 3.5 | 2.1 |
| 101 | Fenoxaprop-P-ethyl | 0.9980 | y = 8375.07006 x – 302.59469 | 0.6 | 2.0 | 93 | 81 | 7.6 | 6.7 |
| 102 | Fenpropathrin | 0.9986 | y = 3475.58555 x + 1117.74226 | 2.0 | 6.0 | 90 | 82 | 5.3 | 6.1 |
| 103 | Fenpropidin | 0.9983 | y = 21482.64167 x – 4957.19254 | 1.0 | 3.0 | 124 | 121 | 3.3 | 4.6 |
| 104 | Fenpyroximate | 0.9972 | y = 9621.41916 x + 707.49421 | 0.6 | 2.0 | 91 | 84 | 2.2 | 3.0 |
| 105 | Fenthion | 0.9987 | y = 2647.29986 x + 2597.84062 | 2.0 | 6.0 | 105 | 92 | 2.0 | 1.8 |
| 106 | Fenthion-sulfone | 0.9979 | y = 7531.63390 x + 6128.19808 | 1.0 | 3.0 | 120 | 114 | 2.6 | 3.5 |
| 107 | Fenthion-sulfoxide | 0.9980 | y = 11396.27020 x + 4906.50514 | 0.6 | 2.0 | 114 | 108 | 4.5 | 0.5 |
| 108 | Fipronil | 0.9986 | y = 951.09224 x + 502.26602 | 2.0 | 6.0 | 111 | 94 | 3.8 | 2.4 |
| 109 | Fipronil-sulfone | 0.9988 | y = 991.86559 x + 1340.80443 | 2.0 | 6.0 | 100 | 84 | 4.2 | 3.5 |
| 110 | Flonicamid | 0.9989 | y = 1113.56397 x + 1251.70089 | 1.0 | 3.0 | 116 | 109 | 6.3 | 5.9 |
| 111 | Florasulam | 0.9995 | y = 2652.55912 x + 268.72463 | 0.6 | 2.0 | 128 | 122 | 4.3 | 2.2 |
| 112 | Fluazifop-butyl | 0.9983 | y = 12427.66861 x + 2589.97183 | 1.0 | 3.0 | 105 | 95 | 5.7 | 4.2 |
| 113 | Flucetosulfuron | 0.9992 | y = 942.45726 x – 157.69566 | 1.0 | 3.0 | 114 | 111 | 3.2 | 5.1 |
| 114 | Flucythrinate | 0.9996 | y = 3592.95543 x – 1367.73973 | 2.0 | 6.0 | 97 | 88 | 2.7 | 0.6 |
| 115 | Fludioxonil | 0.9977 | y = 590.45821 x + 986.28329 | 2.0 | 6.0 | 102 | 96 | 5.1 | 3.8 |
| 116 | Flufenoxuron | 0.9988 | y = 1579.00714 x + 530.64600 | 1.0 | 3.0 | 100 | 85 | 3.1 | 2.6 |
| 117 | Flufiprole | 0.9993 | y = 459.50468 x + 1533.89721 | 3.0 | 10.0 | 118 | 99 | 3.8 | 3.2 |
| 118 | Flumetsulam | 0.9992 | y = 2745.96825 x + 2924.07971 | 1.0 | 3.0 | 128 | 123 | 3.1 | 4.2 |
| 119 | Flumorph | 0.9983 | y = 2845.84073 x – 1275.00529 | 1.0 | 3.0 | 117 | 109 | 2.9 | 1.7 |
| 120 | Fluopicolide | 0.9980 | y = 327.55033 x + 1268.55618 | 3.0 | 10.0 | 114 | 103 | 1.9 | 0.7 |
| 121 | Fluopyram | 0.9991 | y = 1091.73963 x + 137.64429 | 1.0 | 3.0 | 115 | 109 | 3.9 | 2.8 |
| 122 | Fluoroglycofen-ethyl | 0.9973 | y = 2842.67605 x – 580.81738 | 2.0 | 6.0 | 107 | 95 | 4.5 | 6.5 |
| 123 | Fluroxypyr | 0.9994 | y = 1453.28242 x + 6862.96073 | 5.0 | 15.0 | 82 | 72 | 1.9 | 2.7 |
| 124 | Fluroxypyr-meptyl | 0.9992 | y = 1648.98451 x – 150.83222 | 2.0 | 6.0 | 87 | 77 | 5.9 | 3.3 |
| 125 | Flusilazole | 0.9981 | y = 5148.23193 x + 6902.29399 | 1.0 | 3.0 | 100 | 81 | 1.9 | 2.5 |
| 126 | Fluthiacet-methyl | 0.9981 | y = 3661.39822 x – 1621.09958 | 0.6 | 2.0 | 112 | 103 | 1.3 | 2.3 |
| 127 | Flutolanil | 0.9987 | y = 7754.24390 x + 10784.50959 | 1.0 | 3.0 | 115 | 105 | 0.8 | 2.8 |
| 128 | Flutriafol | 0.9976 | y = 5467.27385 x + 948.79740 | 1.0 | 3.0 | 103 | 110 | 3.6 | 4.6 |
| 129 | Fomesafen | 0.9992 | y = 544.21875 x – 617.49337 | 2.0 | 6.0 | 116 | 109 | 6.5 | 5.3 |
| 130 | Fonofos | 0.9995 | y = 790.76533 x + 451.95043 | 3.0 | 10.0 | 90 | 83 | 2.7 | 3.4 |
| 131 | Forchlorfenuron | 0.9996 | y = 3177.56861 x + 208.85958 | 0.6 | 2.0 | 85 | 102 | 2.6 | 0.6 |
| 132 | Fosthiazate | 0.9978 | y = 6812.49086 x + 3536.91927 | 0.6 | 2.0 | 108 | 100 | 3.2 | 2.9 |
| 133 | Halosulfuron-methyl | 0.9985 | y = 977.28094 x – 192.33470 | 1.0 | 3.0 | 119 | 114 | 2.8 | 2.1 |
| 134 | Haloxyfop-methyl | 0.9985 | y = 6439.79528 x – 1862.74508 | 0.6 | 2.0 | 114 | 102 | 1.0 | 0.6 |
| 135 | Hexaconazole | 0.9986 | y = 2109.21961 x + 413.35471 | 1.0 | 3.0 | 102 | 78 | 2.7 | 2.0 |
| 136 | Hexaflumuron | 0.9990 | y = 502.53412 x – 176.14770 | 3.0 | 10.0 | 96 | 100 | 4.1 | 2.2 |
| 137 | Hexazinone | 0.9987 | y = 11074.77160 x + 20086.84162 | 1.0 | 3.0 | 103 | 109 | 3.8 | 1.9 |
| 138 | Hexythiazox | 0.9988 | y = 2696.16151 x – 1227.12833 | 2.0 | 6.0 | 83 | 91 | 4.3 | 5.8 |
| 139 | Hymexazol | 0.9982 | y = 132.34117 x + 234.90770 | 3.0 | 10.0 | 98 | 108 | 9.8 | 10.8 |
| 140 | Imazalil | 0.9979 | y = 9844.96062 x + 3576.24567 | 0.6 | 2.0 | 107 | 84 | 2.6 | 1.3 |
| 141 | Imazapic | 0.9988 | y = 9561.47292 x + 4446.89328 | 1.0 | 3.0 | 98 | 89 | 8.4 | 7.7 |
| 142 | Imazaquin | 0.9985 | y = 12771.46975 x + 4682.43268 | 0.6 | 2.0 | 117 | 106 | 3.5 | 2.1 |
| 143 | Imazethapyr | 0.9990 | y = 9791.91387 x – 5092.90674 | 1.0 | 3.0 | 119 | 108 | 0.7 | 1.7 |
| 144 | Imibenconazole | 0.9986 | y = 1184.75283 x + 1419.00927 | 2.0 | 6.0 | 70 | 72 | 4.0 | 5.2 |
| 145 | Imidacloprid | 0.9984 | y = 2273.72466 x + 2952.21203 | 1.0 | 3.0 | 119 | 115 | 2.9 | 3.6 |
| 146 | Indoxacarb | 0.9993 | y = 2822.15844 x – 1218.75317 | 1.0 | 3.0 | 117 | 108 | 2.5 | 4.3 |
| 147 | Iprobenfos | 0.9983 | y = 3258.79915 x + 3323.82724 | 1.0 | 3.0 | 103 | 98 | 3.3 | 2.2 |
| 148 | Isazofos | 0.9984 | y = 7049.37925 x + 1692.62059 | 0.6 | 2.0 | 112 | 107 | 1.5 | 2.6 |
| 149 | Isoprocarb | 0.9972 | y = 1186.94732 x + 4478.25224 | 10.0 | 30.0 | 110 | 103 | 7.0 | 5.0 |
| 150 | Isoprothiolane | 0.9989 | y = 4361.40517 x – 1862.48132 | 1.0 | 3.0 | 117 | 110 | 1.1 | 3.1 |
| 151 | Isoproturon | 0.9974 | y = 11094.73572 x + 5401.68440 | 0.6 | 2.0 | 111 | 105 | 1.2 | 3.2 |
| 152 | Ivermectin | 0.9990 | y = 779.89382 x – 476.02104 | 3.0 | 10.0 | 102 | 111 | 12.5 | 11.5 |
| 153 | Kasugamycin | 0.9983 | y = 25.77401 x – 42.92681 | 5.0 | 15.0 | 73 | 104 | 1.9 | 9.6 |
| 154 | Kresoxim-methyl | 0.9981 | y = 1515.82251 x – 22.76672 | 2.0 | 6.0 | 102 | 98 | 8.3 | 7.6 |
| 155 | Lactofen | 0.9983 | y = 3307.86576 x – 626.29677 | 1.0 | 3.0 | 103 | 97 | 4.8 | 3.5 |
| 156 | Lufenuron | 0.9988 | y = 474.73393 x + 93.64650 | 3.0 | 10.0 | 89 | 94 | 3.4 | 2.9 |
| 157 | Malathion | 0.9985 | y = 4645.73651 x – 3140.11345 | 1.0 | 3.0 | 117 | 106 | 4.3 | 3.9 |
| 158 | Maleic hydrazide | 0.9987 | y = 168.03948 x + 324.23157 | 2.0 | 6.0 | 135 | 127 | 12.6 | 10.8 |
| 159 | Mandipropamid | 0.9989 | y = 796.88850 x – 80.94004 | 2.0 | 6.0 | 113 | 106 | 2.8 | 4.6 |
| 160 | Mefenacet | 0.9989 | y = 11088.97283 x + 2657.28981 | 0.6 | 2.0 | 112 | 108 | 3.1 | 4.6 |
| 161 | Mepronil | 0.9984 | y = 8453.88413 x + 14772.78170 | 1.0 | 3.0 | 108 | 101 | 1.6 | 2.5 |
| 162 | Meptyldinocap-phenol | 0.9989 | y = 3156.33533 x + 1527.62324 | 3.0 | 10.0 | 108 | 96 | 2.6 | 3.3 |
| 163 | Mesosulfuron-methyl | 0.9983 | y = 931.04563 x + 879.26997 | 1.0 | 3.0 | 120 | 117 | 6.3 | 8.2 |
| 164 | Mesotrione | 0.9987 | y = 406.89832 x + 65.55084 | 2.0 | 6.0 | 119 | 96 | 7.4 | 8.8 |
| 165 | Metaflumizone | 0.9993 | y = 958.47618 x + 685.00746 | 2.0 | 6.0 | 105 | 100 | 3.6 | 1.6 |
| 166 | Metalaxyl | 0.9985 | y = 10742.05192 x + 5223.86146 | 2.0 | 6.0 | 115 | 110 | 3.2 | 1.2 |
| 167 | Metamifop | 0.9984 | y = 7010.24242 x + 5902.10769 | 1.0 | 3.0 | 113 | 107 | 1.4 | 2.3 |
| 168 | Metamitron | 0.9980 | y = 9247.37244 x + 4741.63386 | 0.6 | 2.0 | 94 | 85 | 3.7 | 2.5 |
| 169 | Metazachlor | 0.9973 | y = 3599.69885 x + 2691.28432 | 1.0 | 3.0 | 112 | 104 | 4.3 | 2.9 |
| 170 | Methamidophos | 0.9988 | y = 642.11823 x – 534.12940 | 2.0 | 6.0 | 77 | 85 | 7.2 | 8.0 |
| 171 | Methidathion | 0.9986 | y = 1832.63594 x + 27.10519 | 1.0 | 3.0 | 112 | 94 | 7.3 | 6.1 |
| 172 | Methoxyfenozide | 0.9987 | y = 2893.03300 x + 2344.52811 | 2.0 | 6.0 | 119 | 116 | 3.1 | 9.4 |
| 173 | Metolachlor | 0.9986 | y = 6690.70274 x + 10741.80144 | 1.0 | 3.0 | 107 | 87 | 3.9 | 1.5 |
| 174 | Metribuzin | 0.9983 | y = 5623.84405 x + 4507.39571 | 2.0 | 6.0 | 111 | 114 | 5.1 | 1.7 |
| 175 | Metsulfuron-methyl | 0.9989 | y = 2038.56184 x – 1237.98485 | 1.0 | 3.0 | 122 | 119 | 4.6 | 3.1 |
| 176 | Molinate | 0.9992 | y = 796.33439 x – 212.38378 | 2.0 | 6.0 | 107 | 113 | 2.2 | 3.0 |
| 177 | Monocrotophos | 0.9983 | y = 2364.92593 x + 968.77953 | 1.0 | 3.0 | 106 | 98 | 5.6 | 6.5 |
| 178 | Myclobutanil | 0.9980 | y = 3234.30566 x + 4851.42195 | 1.0 | 3.0 | 109 | 115 | 1.6 | 3.6 |
| 179 | Nicosulfuron | 0.9994 | y = 1762.02579 x + 316.65542 | 1.0 | 3.0 | 114 | 118 | 7.4 | 5.6 |
| 180 | Nitenpyram | 0.9992 | y = 5025.47869 x + 4080.50649 | 0.6 | 2.0 | 91 | 100 | 3.9 | 2.9 |
| 181 | Novaluron | 0.9987 | y = 943.90520 x – 665.42888 | 2.0 | 6.0 | 117 | 109 | 5.7 | 3.3 |
| 182 | Omethoate | 0.9984 | y = 4392.66314 x – 3257.66393 | 1.0 | 3.0 | 83 | 77 | 4.1 | 3.0 |
| 183 | Orthosulfamuron | 0.9980 | y = 152.19479 x + 413.52217 | 3.0 | 10.0 | 89 | 97 | 3.6 | 4.5 |
| 184 | Oxadiargyl | 0.9993 | y = 1731.81150 x + 3104.70287 | 2.0 | 6.0 | 115 | 105 | 5.1 | 2.5 |
| 185 | Oxadiazon | 0.9985 | y = 3027.87184 x + 2233.59722 | 1.0 | 3.0 | 103 | 89 | 3.5 | 2.1 |
| 186 | Oxadixyl | 0.9991 | y = 5939.51396 x – 249.90128 | 1.0 | 3.0 | 113 | 108 | 6.7 | 7.2 |
| 187 | Oxaziclomefone | 0.9991 | y = 6077.03755 x + 3892.44035 | 1.0 | 3.0 | 106 | 99 | 1.6 | 2.5 |
| 188 | Oxydemeton-methyl | 0.9991 | y = 6212.49026 x + 139.11523 | 0.6 | 2.0 | 95 | 103 | 0.6 | 2.4 |
| 189 | Paclobutrazol | 0.9983 | y = 4108.11316 x + 7130.13197 | 1.0 | 3.0 | 100 | 91 | 1.1 | 3.0 |
| 190 | Parathion | 0.9990 | y = 367.54637 x + 3.68093 | 2.0 | 6.0 | 85 | 101 | 10.5 | 12.4 |
| 191 | Penconazole | 0.9981 | y = 3300.78964 x + 2896.54990 | 2.0 | 6.0 | 107 | 97 | 0.6 | 2.6 |
| 192 | Pendimethalin | 0.9989 | y = 897.05504 x + 273.56087 | 3.0 | 10.0 | 87 | 99 | 2.9 | 3.3 |
| 193 | Penoxsulam | 0.9985 | y = 6039.79875 x + 1057.64189 | 0.6 | 2.0 | 131 | 125 | 3.9 | 4.7 |
| 194 | Permethrin | 0.9992 | y = 820.30748 x – 1632.59817 | 5.0 | 15.0 | 73 | 83 | 8.2 | 9.3 |
| 195 | Phenamacril | 0.9985 | y = 230.82294 x + 1557.73708 | 10.0 | 30.0 | 71 | 94 | 6.4 | 8.5 |
| 196 | Phenmedipham | 0.9974 | y = 3476.12866 x + 4195.93829 | 2.0 | 6.0 | 118 | 110 | 8.6 | 7.0 |
| 197 | Phenthoate | 0.9989 | y = 3586.61928 x + 1541.72984 | 2.0 | 6.0 | 102 | 109 | 1.5 | 3.5 |
| 198 | Phorate-sulfone | 0.9976 | y = 2777.60576 x – 782.50165 | 1.0 | 3.0 | 93 | 98 | 6.9 | 8.3 |
| 199 | Phorate-sulfoxide | 0.9991 | y = 4534.92573 x + 3543.53399 | 1.0 | 3.0 | 114 | 110 | 3.1 | 1.1 |
| 200 | Phosalone | 0.9982 | y = 3710.73996 x + 2961.88759 | 1.0 | 3.0 | 104 | 100 | 5.9 | 4.7 |
| 201 | Phosfolan | 0.9992 | y = 8787.32035 x + 6600.00354 | 1.0 | 3.0 | 71 | 79 | 2.6 | 3.9 |
| 202 | Phosfolan-methyl | 0.9985 | y = 4998.03275 x – 2085.14230 | 0.6 | 2.0 | 112 | 109 | 2.4 | 3.5 |
| 203 | Phosmet | 0.9935 | y = 885.79209 x – 3370.30448 | 5.0 | 15.0 | 112 | 105 | 4.7 | 3.5 |
| 204 | Phosphamidon | 0.9981 | y = 10629.49012 x + 193.36301 | 0.6 | 2.0 | 118 | 110 | 5.1 | 1.1 |
| 205 | Phoxim | 0.9985 | y = 2430.86561 x + 1782.61534 | 2.0 | 6.0 | 100 | 91 | 6.0 | 4.5 |
| 206 | Picoxystrobin | 0.9989 | y = 3552.45224 x + 1204.30477 | 1.0 | 3.0 | 119 | 108 | 1.0 | 2.9 |
| 207 | Pinoxaden | 0.9992 | y = 5932.39305 x – 3904.82245 | 2.0 | 6.0 | 111 | 105 | 0.7 | 2.6 |
| 208 | Piperonyl-butoxide | 0.9982 | y = 9446.70327 x + 6248.25472 | 1.0 | 3.0 | 99 | 112 | 2.4 | 3.8 |
| 209 | Pirimicarb | 0.9986 | y = 12100.72433 x + 6309.58079 | 2.0 | 6.0 | 115 | 108 | 0.8 | 2.7 |
| 210 | Pirimiphos-methyl | 0.9983 | y = 7756.46048 x + 698.75455 | 1.0 | 3.0 | 113 | 102 | 3.9 | 2.9 |
| 211 | Pretilachlor | 0.9989 | y = 9239.99749 x – 1397.85982 | 1.0 | 3.0 | 108 | 92 | 2.7 | 1.1 |
| 212 | Prochloraz | 0.9996 | y = 3410.62458 x – 163.58482 | 1.0 | 3.0 | 83 | 97 | 5.9 | 6.9 |
| 213 | Profenofos | 0.9991 | y = 4088.21865 x – 1665.04594 | 1.0 | 3.0 | 97 | 83 | 4.7 | 3.1 |
| 214 | Prometryn | 0.9993 | y = 16152.56549 x + 2450.76437 | 0.6 | 2.0 | 115 | 111 | 2.4 | 3.4 |
| 215 | Propachlor | 0.9985 | y = 3576.19695 x – 815.58966 | 1.0 | 3.0 | 99 | 109 | 6.1 | 4.5 |
| 216 | Propamocarb | 0.9997 | y = 8126.62291 x – 1868.85387 | 0.6 | 2.0 | 64 | 70 | 3.1 | 4.0 |
| 217 | Propanil | 0.9986 | y = 1156.82672 x + 2256.84127 | 1.0 | 3.0 | 112 | 89 | 1.8 | 2.4 |
| 218 | Propargite | 0.9985 | y = 5410.94891 x – 1087.49713 | 1.0 | 3.0 | 98 | 81 | 3.7 | 1.4 |
| 219 | Propiconazole | 0.9992 | y = 3793.98750 x + 2541.03686 | 1.0 | 3.0 | 109 | 93 | 4.0 | 2.5 |
| 220 | Propyzamide | 0.9988 | y = 1571.37151 x + 2071.41470 | 1.0 | 3.0 | 114 | 104 | 2.9 | 1.6 |
| 221 | Prothioconazole-desthiometabolite | 0.9977 | y = 631.83720 x – 23.19560 | 2.0 | 6.0 | 78 | 86 | 2.6 | 0.6 |
| 222 | Pymetrozine | 0.9988 | y = 2824.71526 x + 3561.27575 | 1.0 | 3.0 | 78 | 98 | 2.8 | 7.5 |
| 223 | Pyraclostrobin | 0.9990 | y = 9572.28961 x – 2232.59519 | 0.6 | 2.0 | 113 | 101 | 1.4 | 2.3 |
| 224 | Pyraflufen-ethyl | 0.9985 | y = 3424.32617 x + 1390.15496 | 1.0 | 3.0 | 103 | 91 | 2.9 | 4.3 |
| 225 | Pyrametostrobin | 0.9981 | y = 1480.18946 x + 1528.98253 | 2.0 | 6.0 | 116 | 109 | 3.4 | 1.4 |
| 226 | Pyraoxystrobin | 0.9983 | y = 600.40386 x + 595.26587 | 3.0 | 10.0 | 99 | 82 | 1.9 | 2.7 |
| 227 | Pyrazosulfuron-ethyl | 0.9984 | y = 2165.73263 x + 1696.33509 | 1.0 | 3.0 | 119 | 109 | 9.2 | 7.1 |
| 228 | Pyrethrin I | 0.9987 | y = 1223.12170 x + 491.89540 | 5.0 | 15.0 | 98 | 87 | 3.6 | 1.8 |
| 229 | Pyrethrin II | 0.9989 | y = 687.63096 x + 953.25604 | 5.0 | 15.0 | 94 | 90 | 6.1 | 3.4 |
| 230 | Pyridaben | 0.9981 | y = 6128.36642 x + 8963.47920 | 2.0 | 6.0 | 83 | 91 | 4.1 | 5.5 |
| 231 | Pyriftalid | 0.9985 | y = 16191.12735 x + 7068.53688 | 0.6 | 2.0 | 118 | 110 | 3.1 | 1.0 |
| 232 | Pyrimethanil | 0.9995 | y = 4407.72479 x + 238.68408 | 1.0 | 3.0 | 119 | 100 | 4.1 | 0.9 |
| 233 | Pyriproxyfen | 0.9976 | y = 11787.96771 x – 303.53336 | 0.6 | 2.0 | 91 | 87 | 3.9 | 3.0 |
| 234 | Quinalphos | 0.9985 | y = 6663.95159 x + 9066.77116 | 2.0 | 6.0 | 109 | 101 | 2.0 | 0.9 |
| 235 | Quinoxyfen | 0.9995 | y = 4642.62634 x – 1695.28299 | 0.6 | 2.0 | 78 | 95 | 4.1 | 4.9 |
| 236 | Quizalofop-ethyl | 0.9992 | y = 4086.22373 x + 5748.85356 | 3.0 | 10.0 | 96 | 85 | 5.8 | 4.2 |
| 237 | Rimsulfuron | 0.9980 | y = 454.66428 x + 36.26931 | 2.0 | 6.0 | 103 | 84 | 8.5 | 7.0 |
| 238 | Rotenone | 0.9983 | y = 3912.24219 x + 4952.47425 | 1.0 | 3.0 | 110 | 103 | 1.7 | 1.6 |
| 239 | Saflufenacil | 0.9981 | y = 624.74102 x + 149.28645 | 1.0 | 3.0 | 120 | 117 | 4.1 | 5.3 |
| 240 | Sethoxydim | 0.9950 | y = 6049.13007 x + 11908.29540 | 1.0 | 3.0 | 118 | 105 | 2.9 | 1.7 |
| 241 | Simazine | 0.9971 | y = 3351.91589 x + 141.30166 | 1.0 | 3.0 | 103 | 92 | 0.7 | 2.7 |
| 242 | Simetryn | 0.9984 | y = 13090.64064 x + 6574.11071 | 0.6 | 2.0 | 99 | 105 | 3.1 | 1.2 |
| 243 | Spinetoram A | 0.9982 | y = 7297.15987 x + 170.17315 | 3.0 | 10.0 | 119 | 109 | 3.8 | 5.5 |
| 244 | Spinosad A | 0.9988 | y = 5688.19065 x – 925.78912 | 2.0 | 6.0 | 110 | 107 | 6.5 | 4.4 |
| 245 | Spinosad D | 0.9994 | y = 5257.02869 x + 1089.07406 | 2.0 | 6.0 | 113 | 103 | 3.9 | 5.7 |
| 246 | Spirodiclofen | 0.9988 | y = 1717.90968 x + 302.17013 | 2.0 | 6.0 | 90 | 81 | 5.9 | 4.4 |
| 247 | Spirotetramat | 0.9994 | y = 7605.91730 x – 2922.57627 | 1.0 | 3.0 | 116 | 111 | 2.4 | 3.6 |
| 248 | Spirotetramat-enol | 0.9976 | y = 5712.51916 x + 8695.07108 | 1.0 | 3.0 | 106 | 113 | 4.9 | 6.1 |
| 249 | Sulcotrione | 0.9999 | y = 1335.16776 x + 930.72112 | 2.0 | 6.0 | 111 | 101 | 3.1 | 1.0 |
| 250 | Sulfotep | 0.9990 | y = 6079.57447 x – 962.52875 | 2.0 | 6.0 | 109 | 101 | 4.3 | 2.1 |
| 251 | Sulfoxaflor | 0.9984 | y = 1116.55659 x + 609.30258 | 3.0 | 10.0 | 88 | 96 | 3.2 | 6.6 |
| 252 | tau-Fluvalinate | 0.9979 | y = 1822.85474 x – 1285.60950 | 2.0 | 6.0 | 71 | 103 | 7.7 | 8.4 |
| 253 | Tebuconazole | 0.9983 | y = 3444.50789 x + 7052.11239 | 1.0 | 3.0 | 111 | 80 | 2.7 | 2.0 |
| 254 | Tebufenozide | 0.9994 | y = 1823.23982 x + 2158.77755 | 3.0 | 10.0 | 109 | 115 | 2.0 | 3.7 |
| 255 | Teflubenzuron | 0.9989 | y = 482.79637 x + 244.86934 | 3.0 | 10.0 | 112 | 90 | 4.6 | 3.7 |
| 256 | Terbufos-sulfone | 0.9991 | y = 1715.00248 x + 466.29663 | 1.0 | 3.0 | 119 | 105 | 2.5 | 4.2 |
| 257 | Terbufos-sulfoxide | 0.9992 | y = 4312.88282 x + 2997.57398 | 1.0 | 3.0 | 114 | 107 | 3.8 | 1.7 |
| 258 | Terbuthylazine | 0.9988 | y = 7070.42434 x – 3486.60717 | 0.6 | 2.0 | 109 | 97 | 2.0 | 0.3 |
| 259 | Thiabendazole | 0.9988 | y = 11815.36074 x + 10033.49168 | 1.0 | 3.0 | 84 | 92 | 3.9 | 5.4 |
| 260 | Thiacloprid | 0.9989 | y = 6671.65303 x + 2669.02150 | 0.6 | 2.0 | 82 | 96 | 6.2 | 7.2 |
| 261 | Thiamethoxam | 0.9984 | y = 2046.06774 x + 2464.34038 | 1.0 | 3.0 | 120 | 105 | 3.9 | 2.5 |
| 262 | Thidiazuron | 0.9974 | y = 1164.42630 x + 1190.98291 | 1.0 | 3.0 | 71 | 94 | 8.9 | 11.8 |
| 263 | Thifensulfuron-methyl | 0.9971 | y = 1865.13321 x – 649.54646 | 0.6 | 2.0 | 121 | 112 | 4.3 | 5.9 |
| 264 | Thifluzamide | 0.9972 | y = 121.77652 x + 177.44924 | 5.0 | 15.0 | 93 | 105 | 5.1 | 4.8 |
| 265 | Thiobencarb | 0.9974 | y = 2897.95098 x + 801.07059 | 2.0 | 6.0 | 102 | 88 | 1.3 | 3.3 |
| 266 | Thiodicarb | 0.9994 | y = 2309.88792 x – 2582.80540 | 3.0 | 10.0 | 91 | 81 | 4.5 | 3.2 |
| 267 | Thiophanate-methyl | 0.9995 | y = 8334.44038 x – 6735.22671 | 2.0 | 6.0 | 96 | 86 | 1.7 | 2.5 |
| 268 | Tolclofos-methyl | 0.9989 | y = 1044.79269 x + 844.74323 | 2.0 | 6.0 | 108 | 94 | 2.2 | 6.0 |
| 269 | Tolfenpyrad | 0.9984 | y = 3182.90311 x + 1356.67905 | 2.0 | 6.0 | 105 | 90 | 3.0 | 0.8 |
| 270 | Tolylfluanid | 0.9971 | y = 948.08116 x + 1931.61979 | 2.0 | 6.0 | 98 | 82 | 4.8 | 3.7 |
| 271 | Triadimefon | 0.9984 | y = 4319.37488 x + 4733.32266 | 1.0 | 3.0 | 116 | 109 | 3.7 | 2.5 |
| 272 | Triadimenol | 0.9995 | y = 787.05558 x – 62.14173 | 3.0 | 10.0 | 117 | 98 | 2.9 | 1.6 |
| 273 | Triasulfuron | 0.9984 | y = 2554.90642 x + 152.59358 | 0.6 | 2.0 | 119 | 115 | 5.5 | 3.4 |
| 274 | Triazophos | 0.9987 | y = 14774.45526 x – 3290.99178 | 0.6 | 2.0 | 112 | 104 | 2.3 | 1.3 |
| 275 | Tribenuron-methyl | 0.9939 | y = 3882.17934 x + 2624.81987 | 1.0 | 3.0 | 96 | 98 | 1.5 | 0.9 |
| 276 | Trichlorfon | 0.9978 | y = 2173.66903 x + 5521.10845 | 2.0 | 6.0 | 113 | 117 | 6.9 | 3.0 |
| 277 | Tricyclazole | 0.9981 | y = 9745.60529 x – 53.43415 | 0.6 | 2.0 | 101 | 116 | 7.4 | 8.9 |
| 278 | Trifloxystrobin | 0.9981 | y = 10639.79349 x + 8404.96924 | 1.0 | 3.0 | 115 | 95 | 2.9 | 0.7 |
| 279 | Triflumizole | 0.9974 | y = 262.56527 x + 72.10274 | 5.0 | 15.0 | 90 | 77 | 3.2 | 2.7 |
| 280 | Triflumuron | 0.9990 | y = 1738.96248 x + 1454.45344 | 1.0 | 3.0 | 112 | 97 | 3.3 | 2.8 |
| 281 | Trinexapac-ethyl | 0.9986 | y = 2045.60130 x + 3926.32949 | 2.0 | 6.0 | 119 | 115 | 2.8 | 0.8 |
| 282 | Uniconazole | 0.9991 | y = 3753.47849 x + 4249.99529 | 2.0 | 6.0 | 101 | 97 | 2.6 | 3.5 |
| 283 | Vamidothion | 0.9978 | y = 6802.20438 x + 4220.96937 | 1.0 | 3.0 | 118 | 110 | 4.6 | 1.6 |
| 284 | Zoxamide | 0.9993 | y = 2603.74737 x + 3472.78801 | 1.0 | 3.0 | 113 | 80 | 2.7 | 1.8 |

^a^ Limit of detection

^b^ Limit of quantification

^c^ Relative standard deviation

**Figure S1.** Matrix effect (ME) of 284 compounds in blank strawberry, watermelon, melon, peach, and grape extracts during UPLC-QTOF/MS analysis.

**Table S3.** Sampling information of fruits in different districts of Shanghai.

| **Districts** | **Sampling sites (number of samples)** | | | | |
| --- | --- | --- | --- | --- | --- |
|  | **Strawberry**  **(Jan. 2020)** | **Watermelon**  **(Jul. 2019)** | **Melon**  **(Jul. 2019)** | **Peach**  **(Jul. 2019)** | **Grape**  **(Aug. 2020)** |
| Fengxian | 3 Fields (15) | 3 Fields (30) | 3 Fields (30) | 1 Field (10) |  |
| Jiading | 3 Fields (15) |  |  | 2 Field (20) |  |
| Jinshan | 3 Fields (15) |  |  |  |  |
| Pudong | 3 Fields (15) | 2 Fields (20) | 2 Fields (20) | 1 Field (10) | 1 Field (40) |
| Qingpu | 4 Fields (20) |  |  |  |  |

**Table S4.** Average body weight and fruit intake of different age/sex consumer groups in China.

| **Age** | **Sex** | **Body weight (kg)** | **F^a^ (kg/d)** | **LP^b^ (kg/d)** |
| --- | --- | --- | --- | --- |
| 2–4 | Male | 14.1 | 0.0437 | 0.3394 |
|  | Female | 13.4 | 0.0444 | 0.3394 |
| 18–30 | Male | 60.5 | 0.0418 | 0.5102 |
|  | Female | 52.6 | 0.0529 | 0.5102 |
| 60–70 | Male | 61.3 | 0.0338 | 0.5102 |
|  | Female | 54.3 | 0.0348 | 0.5102 |

^a^ Dietary consumption of fruits in different groups

^b^ The large portion of fruit consumption in different groups
